# Bridging radiomics and histopathology: cross-modality consistency of radiomic features from CT to micro-CT in NSCLC

**DOI:** 10.1186/s41747-026-00780-5

**Published:** 2026-10-07

**Authors:** Leonardo Brizzi, Chandra Bortolotto, Raffaella Fiamma Cabini, Francesca Brero, Silvia Megalizzi, Gioacchino D’Ambrosio, Marta Filibian, Roberta Gioia, Paola Falvo, Giulia Maria Stella, Olivia Maria Bottinelli, Alessandro Lascialfari, Lorenzo Preda

**Affiliations:** 1 Department of Radiology, IRCCS Policlinico San Matteo, Pavia, Italy; 2 Department of Clinical, Surgical, Diagnostic and Pediatric Sciences, University of Pavia, Pavia, Italy; 3 Department of Environmental and Prevention Sciences, University of Ferrara, Ferrara, Italy; 4 Department of Physics, University of Pavia, Pavia, Italy; 5 Istituto Nazionale Di Fisica Nucleare, Sezione Di Pavia, Pavia, Italy; 6 Department of Physics, University of Genova, Genoa, Italy; 7 Department of Medical Physics, Azienda Sociosanitaria Ligure 5, La Spezia, Italy; 8 IRCCS Policlinico San Matteo, Pavia, Italy; 9 Biomedical Imaging Laboratory, Centro Grandi Strumenti, University of Pavia, Pavia, Italy; 10 Department of Internal Medicine and Medical Therapeutics, University of Pavia, Pavia, Italy; 11 Unit of Respiratory Diseases, Cardiothoracic and Vascular Department, IRCCS Policlinico San Matteo, Pavia, Italy

**Keywords:** Biomarkers, Carcinoma (non-small-cell lung), Radiomics, Tomography (x-ray computed), x-ray microtomography

## Abstract

**Objective:**

Radiomics extracts quantitative features from medical images that may provide non-invasive biomarkers of tumor biology and heterogeneity. However, few studies have explored radiomics on micro-computed tomography (micro-CT) or its transferability to clinical CT. This study investigates the reproducibility and the potential biological correlation of radiomic features across *in vivo* CT, *ex vivo* CT, and *ex vivo* micro-CT in non-small-cell lung cancer (NSCLC).

**Materials and methods:**

A retrospective cohort of 60 NSCLC patients with preoperative CT scans and surgical specimens was analyzed. *Ex vivo* CT and micro-CT images were acquired and compared with *in vivo* CT using standardized preprocessing, including normalization, resampling, and registration. Tumor regions of interest (ROIs) were semi-automatically segmented, and 93 radiomic features were extracted with Pyradiomics. Cross-modality reproducibility and associations with histotype were evaluated using Spearman correlation, intraclass correlation coefficient, Bland–Altman analysis, and Mann–Whitney *U* test.

**Results:**

Normalization and volume matching improved feature repeatability, with the highest agreement observed in normalized datasets using subsampled ROIs. Four texture-based features consistently showed high reproducibility and potential biological correlation, likely reflecting intratumoral heterogeneity and variation in cell density: DependenceNonUniformity, GrayLevelNonUniformity (GLDM and GLRLM), and Coarseness (NGTDM). These features reliably distinguished adenocarcinoma from squamous cell carcinoma across modalities, confirming their stability and potential as candidate features for transferable biomarkers.

**Conclusion:**

Harmonization and volume standardization are essential for cross-modality radiomic analyses. Stable texture features provide a potential transferable signature bridging micro-CT and clinical CT imaging in NSCLC, supporting their role as candidate features for further investigation in biologically oriented studies.

**Key Points:**

***Question*** Micro-CT serves as an intermediate imaging scale enabling radiomic feature extraction from *ex vivo* NSCLC tumors, allowing direct comparison with *in vivo* and *ex vivo* CT.

***Findings*** Intensity normalization and volume-matched subsampling between *in vivo* CT, *ex vivo* CT, and micro-CT reduced cross-modality feature variability, improving feature reproducibility.

***Relevance statement*** Normalization, volume matching, and cross-modality analysis support the identification of candidate texture features with consistent cross-scale behavior and exploratory associations with NSCLC histotype.

**Graphical Abstract:**

**Figure:**
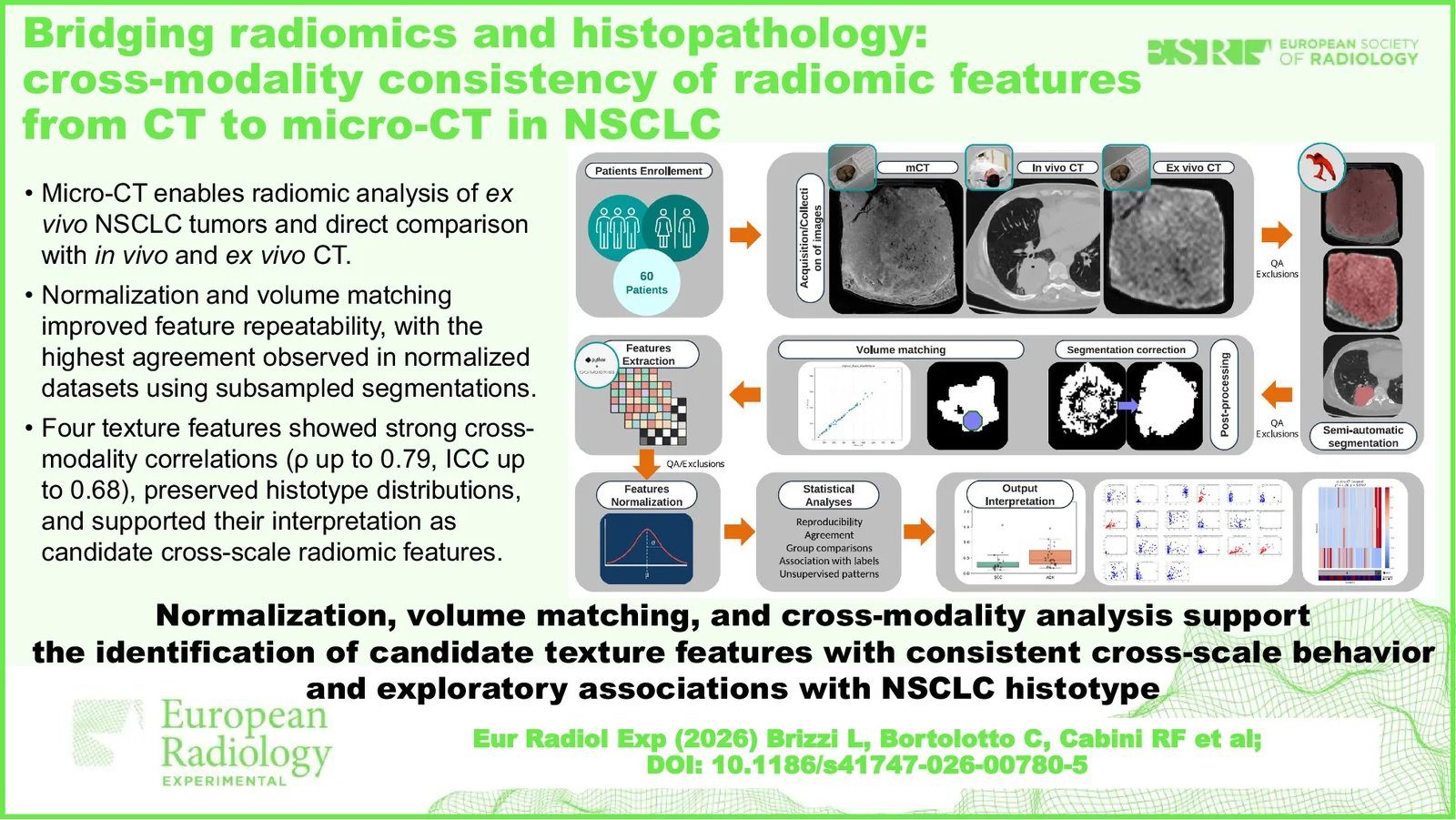

## Background

Radiomics is a rapidly advancing discipline focused on the extraction of high-dimensional quantitative features from medical images, converting these features into non-invasive biomarkers capable of revealing insights into tumor biology, pathology, and potentially treatment outcomes [1–3]. Among its various applications, radiomics has been extensively used to study non-small-cell lung cancer (NSCLC), to enhance diagnostic accuracy, guide treatment planning, and monitor therapeutic responses [4–7].

Unlike traditional biomarker development, which is typically driven ab initio by biology-based hypotheses, radiomic biomarker development is almost entirely data-driven. Without an underlying biological rationale, the “black-box” nature of -omics methods significantly limits their broader use and makes validation particularly challenging. Several efforts to reintroduce biological meaning into radiomics are gaining traction in the field, employing distinct approaches such as genomic correlations, local microscopic pathologic image textures, and macroscopic histopathologic marker expression [8]. However, direct comparisons between histopathology and *in vivo* imaging remain challenging due to differences in spatial scale, resolution, and orientation.

Radiomic features are considered “fragile” biomarkers as they can be influenced by imaging protocol, scanner type, and reconstruction settings [9, 10]. Nevertheless, many of these sources of variability can be mitigated through standardization of image acquisition and preprocessing [11]. For this reason, ongoing efforts aim to standardize imaging acquisition and feature extraction protocols (*e.g*., the Image Biomarker Standardisation Initiative, IBSI) [12].

*Ex vivo* micro-computed tomography (micro-CT) offers histologically relevant resolution while preserving three-dimensional context. Unlike conventional clinical CT, micro-CT can achieve voxel sizes in the micrometer range, enabling visualization of details that are otherwise undetectable with standard CT [13, 14]. This approach may provide an intermediate imaging scale that can bridge the gap between imaging-based radiomics and histopathological validation, potentially allowing the identification of biologically oriented features. In preclinical studies, micro-CT has been successfully used to derive radiomic features comparable to those obtained from other CT platforms. Brown et al showed that, when acquisition parameters are harmonized, micro-CT and cone-beam CT can yield reproducible, tissue-specific feature sets [15].

In this context, the present study aims to: (1) assess the feasibility of performing radiomic analysis on micro-CT images of NSCLC tumor samples; (2) evaluate the consistency of radiomic features across *in vivo* CT, *ex vivo* CT, and *ex vivo* micro-CT, using robust preprocessing steps to mitigate the interscanner variability; and (3) identify candidate features showing consistent cross-modality behavior for exploratory histotype-related analyses.

## Methods

**Fig. 1:**
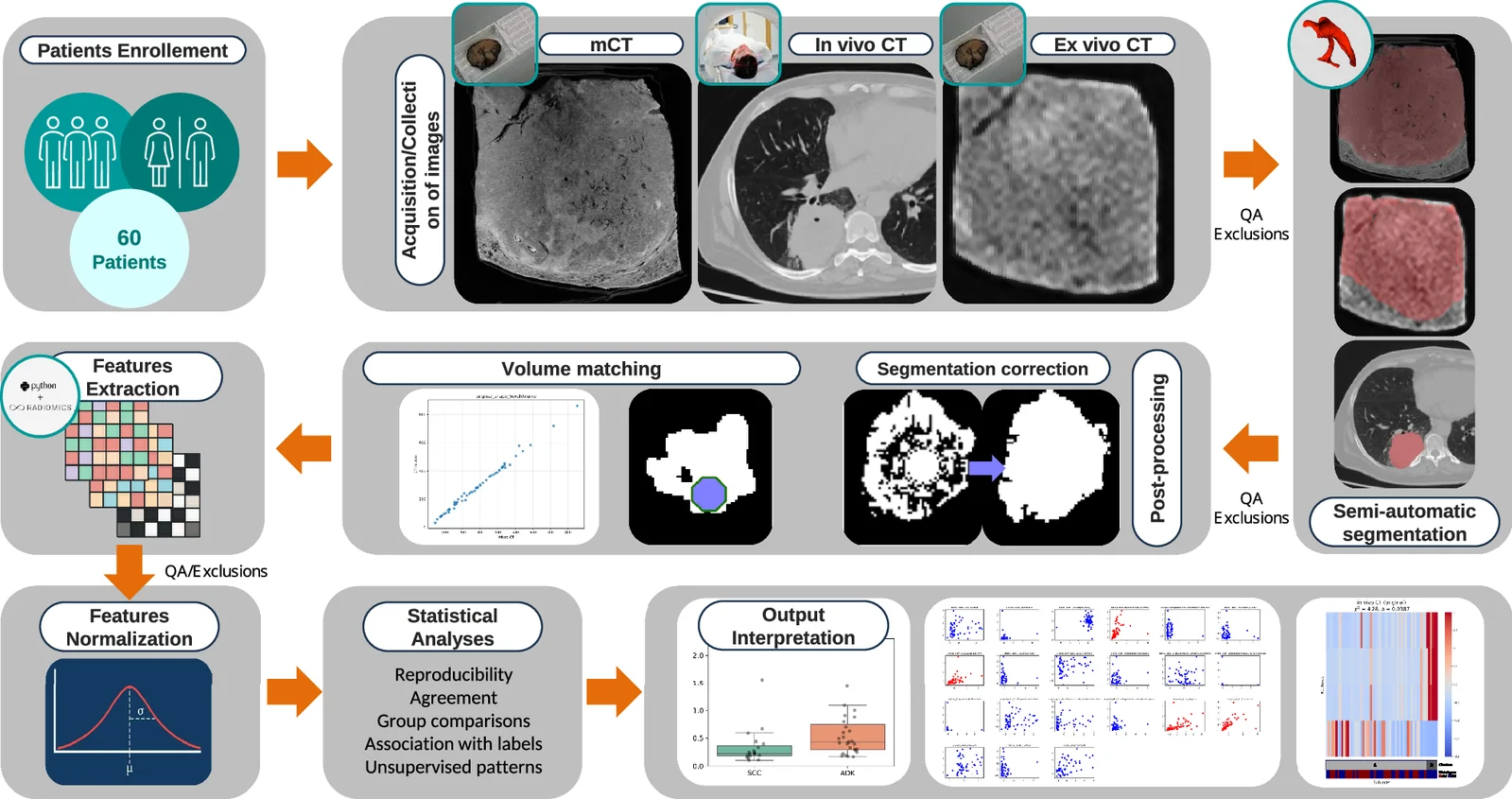
Radiomic analysis workflow. Schematic overview of the study pipeline. After patient enrollment (*n* = 60), *in vivo* CT, *ex vivo* CT, and *ex vivo* micro-CT images were collected. Tumors were segmented semi-automatically, followed by quality assessment (QA) and corrections for segmentation errors. Preprocessing included histogram intensity normalization, volumetric matching between modalities, and post-processing for alignment. Radiomic features were then extracted using IBSI-compliant software (PyRadiomics) and normalized before statistical analysis. Statistical analyses comprised reproducibility and agreement testing (Spearman correlation, intraclass correlation coefficient (ICC), Bland–Altman), group comparisons, and unsupervised clustering. The resulting outputs were used for biological interpretation and feature stability assessment. ADK, Adenocarcinoma; CT, Computed tomography; Micro-CT, Micro-computed tomography; QA, Quality assessment; SCC, Squamous cell carcinoma

The proposed radiomic pipeline is described in Fig. 1.

### Cohort selection

60 patients with NSCLC have been retrospectively enrolled. Inclusion criteria: adult patients diagnosed with NSCLC treated with surgery without previous therapy, availability of surgical specimens, and *in vivo* imaging prior to surgery. Exclusion criteria: inadequate quality of *in vivo* imaging, pre-surgical confounding factors (*e.g*., radiotherapy, chemotherapy, immunotherapy). This study was conducted in accordance with the Declaration of Helsinki and approved by the institutional review board. Informed consent for the retrospective use of imaging data and biological specimens was obtained whenever applicable.

### Image acquisition

**Fig. 2:**
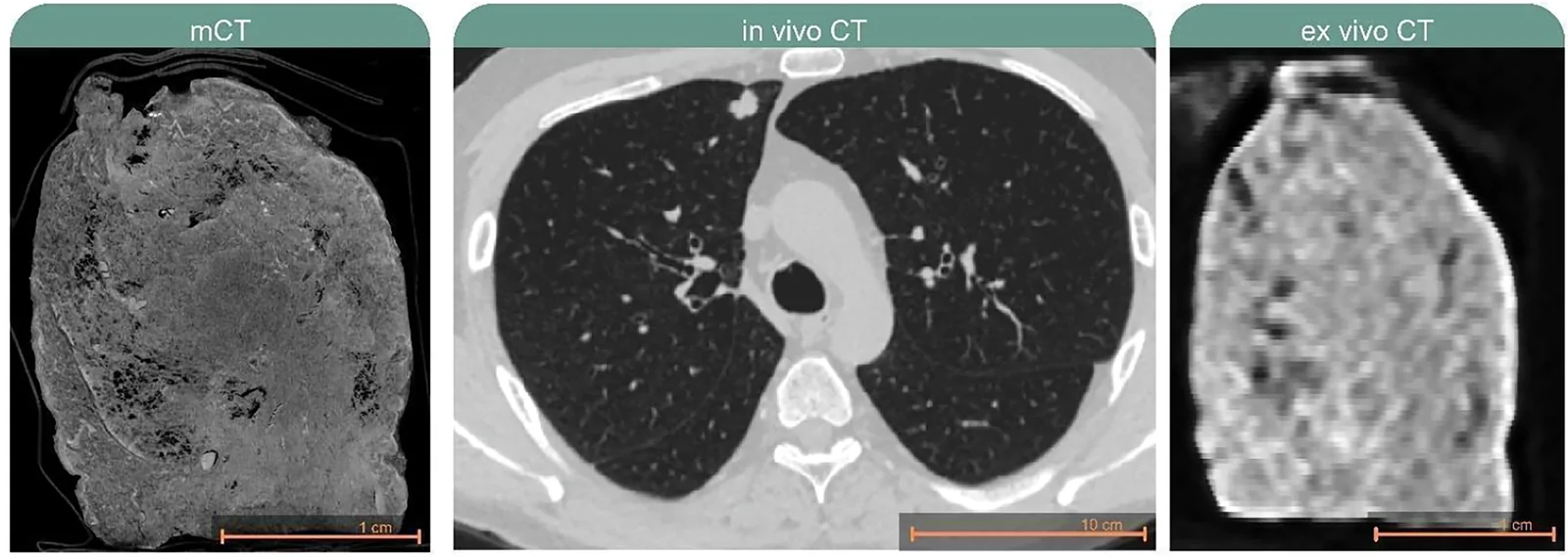
Representative images from the same patient acquired with different imaging modalities used in the study. From left to right: micro-CT, *in vivo* CT, and *ex vivo* CT. The images illustrate the corresponding anatomical regions across modalities, highlighting differences in spatial resolution, field of view, and tissue contrast. Scale bars indicate 1 cm for micro-CT and *ex vivo* CT, and 10 cm for *in vivo* CT. The high-resolution micro-CT image allows visualization of fine structural details, whereas *in vivo* CT provides whole-organ anatomical context. CT, Computed tomography; Micro-CT, Micro-computed tomography

*Ex vivo* conventional CT and micro-CT images of the surgical specimens were acquired, and *in vivo* CT images were retrieved from the Picture Archiving and Communication System archive. Representative axial images from the same patient are shown in Fig. 2. Acquisition parameters for each modality are detailed below.

#### *Ex vivo* micro-CT protocol

Scans were performed with a SkyScan1276 *in vivo* scanner (Bruker®, Kartuizersweg3B.2550, Kontich, Belgium), using a Voltage-Current combination of 40 kVp–200 µA (no x-ray filtering), in step and shoot (circular trajectory) and rotation step of 0.2° over 360° range. Camera binning was set to 1 × 1 with an exposure time of 390 ms and 2 frame averages, yielding 20 µm isotropic pixel size (voxel size 20 × 20 × 20 μm^3^). Reconstruction was performed using the InstaRecon® (SkyScan NRecon software v2.2.0.6), with a Hamming window filter (frequency cutoff = 100%, α = 0.54), misalignment compensation, beam hardening correction of 30%, Gaussian Smoothing Kernel, ring artifacts reduction, defect pixel masking, and setting the limit linear attenuation values to [0, 0.071] mm^-1^.

#### *In vivo* CT protocol

Scans were performed for diagnostic or staging purposes in patients with NSCLC prior to surgery. Due to the retrospective design, images were acquired on different scanners and models. Only scans with a slice thickness ≤ 3 mm were included (voxel size: 0.586–1.367 mm in-plane). Only non-contrasted images were used for the study.

#### *Ex vivo* CT protocol

Scans were performed on a Somatom Go.Top (Siemens®) scanner using spiral acquisition modes, tube voltages of 120 kV, slice thickness of 0.6 mm, 0.3 mm increment, and pitch of 0.8. The field of view was 18 × 10 cm, and the acquisition time was approximately 2 s (voxel size: 0.097 × 0.097 × 0.3 mm³). Image reconstruction was performed with Br40 kernel and ADMIRE iterative reconstruction levels A3.

### Image segmentation and postprocessing

The tumor region of interest (ROI) was segmented by an experienced radiologist, both for CT and micro-CT images, using a validated semi-automatic approach through open-source software ITK-SNAP (http://www.itksnap.org). The micro-CT images were segmented by an experienced radiologist and reviewed in consensus with a pathologist to support tumor contour definition. All datasets underwent preprocessing to minimize acquisition-related variability. First, for all three imaging modalities, segmentation masks were refined through morphological operators to correct minor segmentation errors. Voxel resampling was then performed to standardize spatial resolution and obtain isotropic voxels of 0.58 × 0.58 × 0.58 mm³ for *in vivo* CT, and of 0.097 × 0.097 × 0.097 mm³ for *ex vivo* CT. Gray-level normalization was applied to the ROIs of *in vivo* and *ex vivo* CT to match the median and interquartile range of the corresponding micro-CT ROIs, standardizing intensity distributions across modalities. *Ex vivo* CT images were registered to the micro-CT reference using a semi-automatic procedure based on SimpleITK’s non-rigid B-spline registration, followed by manual correction in cases of misalignment. Tumors were often larger than the paraffin blocks scanned with micro-CT; therefore for *in vivo* CT, images and segmentations, originally including the entire tumor, were reoriented to match the axial orientation of the excised tissue block, and a cylindrical subvolume centered on the tumor was extracted to match the location and volume of the corresponding *ex vivo* CT and micro-CT segmentations (Fig. 1 of the Supplementary Materials) [16–19].

### Radiomic features extraction and statistical analysis

Radiomics features were extracted with Pyradiomics (v.3.0.1) [20], compliant with the IBSI standard [12]. A total of 107 radiomics features were extracted from each ROI, both for micro-CT and conventional CT images. The full list is provided in Supplementary Materials Table 1. Feature values were standardized using z-score normalization to remove scale-dependent effects and allow direct comparison. The shape features were excluded from the analysis to avoid biased results, as the ROIs did not preserve the original tumor geometry, and 93 features were used for the final analyses. All statistical analyses were performed in Python (v3.9), with the significance level set at *p* < 0.05. All *p*-values were corrected for multiple comparisons using the Bonferroni method.

Agreement between imaging modalities (*ex vivo* CT *versus* *in vivo* CT, micro-CT *versus* *in vivo* CT, and micro-CT *versus* *ex vivo* CT) was assessed using, for each feature, Spearman’s rank correlation coefficient (ρ). s. *p*-values were categorized into four significance levels: *p* < 0.001, *p* < 0.01, *p* < 0.05, and *p* ≥ 0.05 (not significant). The intraclass correlation coefficient (ICC) (two-way random-effects model, absolute agreement) was calculated to quantify reproducibility, and Bland–Altman analysis was performed to assess agreement. ICC values were interpreted as poor (< 0.5), moderate (0.5–0.75), good (0.75–0.9), or excellent (≥ 0.9). The average absolute relative difference between modalities (%Change) was computed as |V₁ - V₂| / |V₂| × 100 and expressed as a percentage.

Stable features were identified as those showing *p* ≤ 0.001 across all modality comparisons and were considered candidates for biological association analysis. First-order features were excluded from this selection process, as their agreement may be influenced by the gray-level normalization procedure. Feature stability was primarily assessed using Spearman correlation to evaluate the preservation of monotonic trends across patients between imaging modalities. This choice was motivated by the cross-modality setting, where differences in spatial resolution and intensity distributions limit the applicability of absolute agreement metrics such as the ICC.

Association of stable features with histotypes was evaluated using the Mann–Whitney *U* test, to test for significant differences in feature distributions between adenocarcinoma (ADK) and squamous cell carcinoma (SCC). Unsupervised clustering was performed on both the complete sets of normalized radiomic features and the subset of stable features from CT and micro-CT using the k-means algorithm (*k* = 2). Cluster agreement with histotype categories was evaluated using the χ² test of independence.

## Results

The study cohort consisted of 60 patients (65% male, 35% female) with a mean age of 74.4 ± 7.7 (mean ± standard deviation) years (range 54–91 years). The histological classes were equally distributed between ADK and SCC. All demographic and histopathological characteristics of the cohort are summarized in Table 2 of the Supplementary Materials.

Analyses were performed on two datasets: one with unnormalized gray levels and one after gray-level normalization. Each dataset included three modality comparisons (*ex vivo* CT *versus* *in vivo* CT, micro-CT *versus* *in vivo* CT, and micro-CT *versus* *ex vivo* CT). The mean tumor maximum diameter was 2.97 ± 1.58 cm (range 1.0–7.5 cm); however, specimens were preserved as paraffin-embedded tissue blocks with a maximum dimension of ~2 cm, such that in many cases the entire tumor could not be included for micro-CT acquisition. For this reason, for comparisons involving *in vivo* CT, analyses were repeated on both whole-tumor (Original) and volume-matched (Subsampled) segmentations.

### Normalized datasets cross-modality agreement

The percentage average variation across modalities ranged between 363 and 471%, with higher variability observed for texture features (up to 440%) compared to first-order features (≈320%). For the *ex vivo* CT *versus* *in vivo* CT comparison with ROI subsampling, the following are observed: variation of approximately 373.2% ± 430.6% (mean ± standard deviation); variation of first-order features of approximately 137.84% ± 131.21%. In the same comparison without ROI subsampling, the following variation is observed: for all features, 400.4% ± 425.5% and for first-order features, 305.7% ± 389.0%. Comparing micro-CT and *in vivo* CT, subsampling produces a variation of 363.1% ± 461.2%, and the evaluation of the entire ROI produces a variation of 386.4% ± 558.2%. In this comparison, the variation for first-order features goes from 139.4% ± 146.7% (Subsampled) to 234.3% ± 212.6% (Original). The highest Spearman’s coefficient (ρ) and ICC values are obtained when comparing *ex vivo* CT with subsampled *in vivo* CT [ρ = 0.23 ± 0.31; ICC = 0.23 ± 0.31]. The highest Spearman coefficients are obtained for first-order features (range = 0.51–0.63). Texture features show a ρ between 0.08 and 0.14.

Among the comparisons, those with Subsampled volume (micro-CT *versus* *in vivo* CT Subsampled and *ex vivo* CT *versus* *in vivo* CT Subsampled) and the comparison between micro-CT and *ex vivo* CT show the highest number of significantly associated features (range = 19–20), most of which show high statistical significance (*p* < 0.001). The number of statistically associated features is lower in the remaining comparisons (range = 9–13). Comparisons with subsampled volume show an average ICC among all features ranging from 0.19 to 0.23. In these comparisons, first-order features show a higher average ICC (range = 0.64–0.71, moderate agreement) than textures (range = 0.07–0.13, poor agreement). Comparisons without subsampling show an average ICC of 0.12–0.18 for all features, 0.55–0.62 for first-order features, and 0.02–0.07 for texture features. Results for the normalized datasets are reported in Table 1.

**Table 1:** Summary of results for normalized datasets

Parameter	Group	Comparisons				
		CT *ex vivo* *versus* CT *in vivo* Subsampled	CT *ex vivo* *versus* CT *in vivo* Original	Micro-CT *versus* CT *ex vivo*	Micro-CT *versus* CT *in vivo* Subsampled	Micro-CT *versus* CT *in vivo* Original
%Change	All features	**373.22** ± **430**.**56** (0.00; 3,309.24)	**400.39** ± **425.47** (0.00; 3,470.44)	**471.33** ± **594.60** (5.58; 3,508.88)	**363.09** ± **461.24** (8.98; 3,960.77)	**386.39** ± **558.21** (9.33; 4,870.25)
	First order	**137.84** ± **131.21** (0.00; 439.69)	**305.66** ± **388.96** (0.00; 889.61)	**323.74** ± **808.35** (5.58; 3,508.88)	**139.40** ± **146.72** (8.98; 515.02)	**234.26** ± **212.62** (9.33; 596.63)
	Texture	**429.72** ± **458.04** (132.66; 3,309.24)	**423.12** ± **442.67** (118.78; 3,470.44)	**506.75** ± **531.85** (124.01; 3,406.87)	**416.77** ± **494.43** (104.13; 3,960.77)	**442.90** ± **608.30** (100.78; 4,870.25)
Spearman (ρ)	All features	**0.23** ± **0.31** (-0.26; 1.00)	**0.20** ± **0.28** (-0.37; 1.00)	**0.20** ± **0.32** (-0.22; 0.98)	**0.20** ± **0.33** (-0.27; 0.98)	**0.17** ± **0.29** (-0.29; 0.98)
	First order	**0.60** ± **0.40** (-0.01; 1.00)	**0.58** ± **0.32** (0.14; 1.00)	**0.63** ± **0.38** (-0.03; 0.98)	**0.58** ± **0.37** (0.02; 0.98)	**0.51** ± **0.39** (-0.21; 0.98)
	Texture	**0.14** ± **0.21** (-0.26; 0.66)	**0.10** ± **0.17** (-0.37; 0.40)	**0.10** ± **0.20** (-0.22; 0.78)	**0.11** ± **0.24** (-0.27; 0.82)	**0.08** ± **0.18** (-0.29; 0.49)
Spearman (*p* < 0.05)	All features	21.51% (20)	9.68% (9)	20.43% (19)	21.51% (20)	13.98% (13)
	First order	66.67% (12)	9.68% (9)	66.67% (12)	66.67% (12)	61.11% (11)
	Texture	10.67% (8)	0.00% (0)	9.33% (7)	10.67% (8)	2.67% (2)
Spearman (*p* < 0.01)	All features	19.35% (18)	9.68% (9)	19.35% (18)	20.43% (19)	11.83% (11)
Spearman (*p* < 0.001)	All features	17.20% (16)	9.68% (9)	18.28% (17)	20.43% (19)	9.68% (9)
ICC	All features	**0.23** ± **0.31** (-0.18; 1.00)	**0.18** ± **0.30** (-0.28; 1.00)	**0.19** ± **0.32** (-0.21; 1.00)	**0.20** ± **0.32** (-0.29; 1.00)	**0.12** ± **0.30** (-0.33; 1.00)
	First order	**0.69** ± **0.39** (0.01; 1.00)	**0.62** ± **0.38** (-0.01; 1.00)	**0.71** ± **0.33** (-0.07; 1.00)	**0.64** ± **0.37** (-0.06; 1.00)	**0.55** ± **0.37** (-0.16; 1.00)
	Texture	**0.13** ± **0.16** (-0.18; 0.52)	**0.07** ± **0.15** (-0.28; 0.40)	**0.07** ± **0.15** (-0.21; 0.57)	**0.09** ± **0.20** (-0.29; 0.70)	**0.02** ± **0.17** (-0.33; 0.42)
ICC > 0.5 (moderate-good-excellent)	All features	17.20% (16)	11.83% (11)	15.05% (14)	18.28% (17)	11.83% (11)
	First order	72.22% (13)	61.11% (11)	72.22% (13)	72.22% (13)	61.11% (11)
	Texture	4.00% (3)	0.00% (0)	1.33% (1)	5.33% (4)	0.00% (0)
ICC > 0.75 (good-excellent)	All features	11.83% (11)	9.68% (9)	11.83% (11)	8.60% (8)	6.45% (6)
	First order	61.11% (11)	50.00% (9)	61.11% (11)	44.44% (8)	33.33% (6)
	Texture	0.00% (0)	0.00% (0)	0.00% (0)	0.00% (0)	0.00% (0)
ICC > 0.90 (excellent)	All features	8.60% (8)	8.60% (8)	7.53% (7)	6.45% (6)	5.38% (5)
	First order	44.44% (8)	44.44% (8)	38.89% (7)	33.33% (6)	27.78% (5)
	Texture	0.00% (0)	0.00% (0)	0.00% (0)	0.00% (0)	0.00% (0)

The table reports comparisons between *ex vivo* CT *versus* *in vivo* CT, micro-CT *versus* *ex vivo* CT, and micro-CT *versus* *in vivo* CT, considering CT *in vivo* both with whole-tumor ROIs (Original) and with volume-matched cylindrical ROIs (Subsampled). For each comparison, results are provided for all features combined, as well as for the main families of features analyzed (First order and Texture). Quantitative parameters are expressed as mean ± standard deviation on the first line and (minimum; maximum) on the second line for continuous variables, and as percentage on the first line and (absolute number) on the second line for categorical outcomes. %Change represents the average absolute relative variation in feature values across modalities, Spearman (ρ) indicates the correlation coefficient; Spearman (*p* < 0.05), Spearman (*p* < 0.01), and Spearman (*p* < 0.001) refer to the percentage (and number) of features showing statistically significant correlations at the respective thresholds. Bold values highlight the results considered most informative for the interpretation of the comparisons

*CT* Computed tomography, Micro-CT Micro-computed tomography, *ICC* Intraclass correlation coefficient, *Original* Whole-tumor segmentation, *Subsampled* Volume-matched segmentation, *%Change* Percentage of change

### Non-normalized datasets cross-modality agreement

The average relative variation across modalities ranged between 399.8% and 596.5%, with higher variability observed for first-order features (up to 1,200%) compared to texture features (≈ 450%). The variation is higher than the normalized datasets, for first-order features (> 1,000% *versus* ≈ 320%). Mean ICC values ranged between 0.01 and 0.12 across modality, with no features showing ICC > 0.75, with no good/excellent agreement. Mean Spearman correlation coefficients were low (ρ = 0.05–0.13), and the proportion of significant associations (*p* < 0.05) was below 11%. Even in non-normalized datasets, groups with subsampled ROIs showed better metrics than the original groups (all non-normalized dataset results are in Table S3 of the Supplementary Materials).

**Fig. 3:**
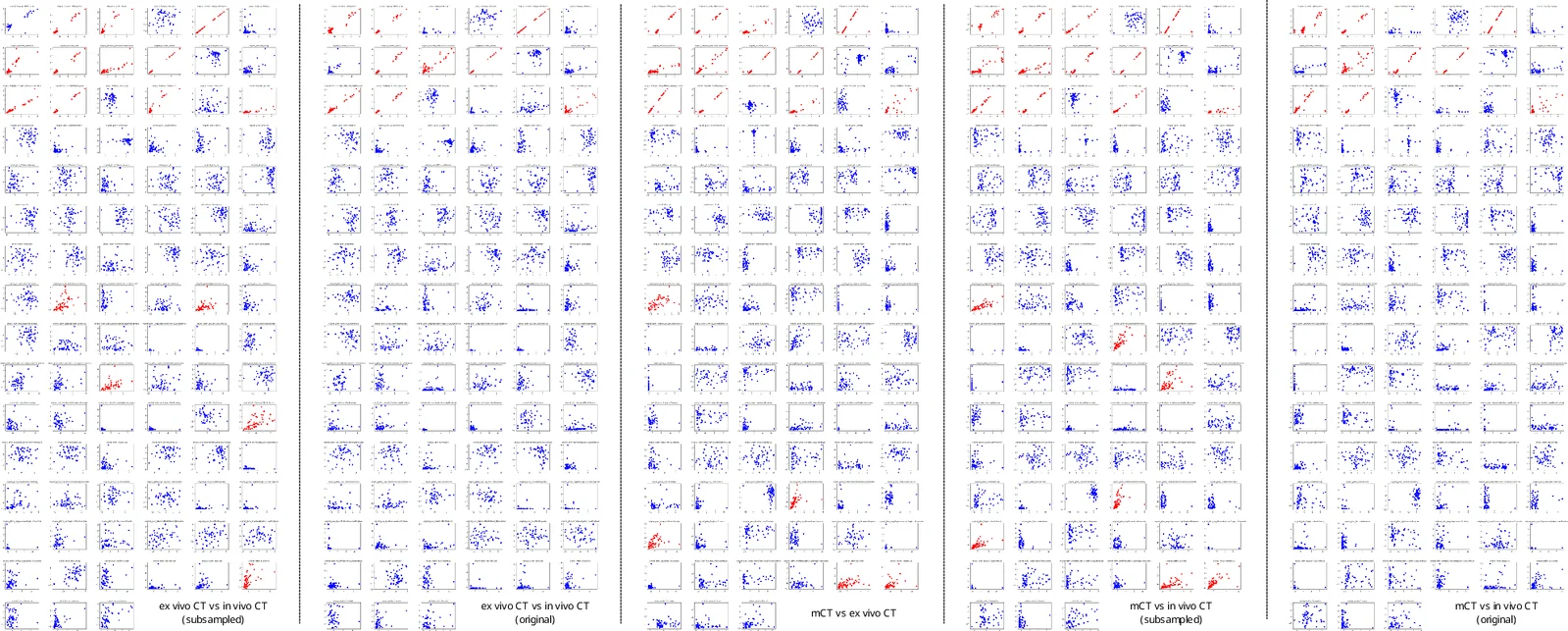
Scatter plot matrices showing pairwise correlations between radiomic features extracted from different imaging modalities after gray-level normalization (normalized dataset). The comparisons include *ex vivo* CT *versus* *in vivo* CT (subsampled), *ex vivo* CT *versus* *in vivo* CT (original), micro-CT *versus* *ex vivo* CT, micro-CT *versus* *in vivo* CT (subsampled), and micro-CT *versus* *in vivo* CT (original). Red points indicate pairs of features showing highly significant correlations (Spearman’s rank correlation coefficient, *p* < 0.001). The plots demonstrate that many first-order features remain consistent across imaging conditions, whereas certain texture features exhibit higher concordance, particularly in the comparisons performed after volume matching. CT, Computed tomography; GLCM, Gray-level co-occurrence matrix; GLDM, Gray-level dependence matrix; GLRLM, Gray-level run length matrix; GLSZM, Gray-level size zone matrix; Micro-CT, Micro-computed tomography; NGTDM, Neighboring gray tone difference matrix; Original, Whole-tumor segmentation; Subsampled, Volume-matched segmentation

It is important to highlight that, since normalization directly aligns the mean and interquartile range of gray-level distributions, the resulting improvement in agreement among first-order features compared to the non-normalized dataset is expected, and these analyses verify the effectiveness of the normalization. However, not all first-order features reached full agreement even in the normalized dataset, reflecting that some histogram characteristics—particularly those describing the overall shape of the distribution—are not explicitly constrained by this procedure (as shown in Fig. 3).

### Features consistency

Feature correlations for normalized datasets are in Fig. 3, and for non-normalized datasets in Fig. 2 of the Supplementary Materials. Bland–Altman analyses are in Figs. 3 and 4 of the Supplementary Materials, and Spearman coefficients are in Figs. 5 and 6 of the Supplementary Materials.

As reported in Fig. 3, some features are stable across the various modalities. Among these, we selected four features that showed high statistical significance (Spearman rank correlation coefficient ρ between 0.579 and 0.792, and *p* < 0.001) in both normalized and non-normalized datasets with subsampled ROIs: original_gldm_DependenceNonUniformity, original_gldm_GrayLevelNonUniformity, original_glrlm_GrayLevelNonUniformity, and original_ngtdm_Coarseness. The metrics of these features are summarized in Table 2 in the manuscript (normalized) and in Table S4 of the Supplementary Materials (non-normalized).

**Table 2:** Metrics of the most stable texture features across modality comparisons in normalized datasets

Dataset/feature	Percentage change	Spearman ρ	Spearman *p*-value	ICC
*Ex vivo* CT *versus* *in vivo* CT (subsampled)				
original_gldm_DependenceNonUniformity	828.33	0.641832	**0.0001**	0.447000
original_gldm_GrayLevelNonUniformity	273.58	0.614417	**0.0001**	0.382000
original_glrlm_GrayLevelNonUniformity	185.55	0.655056	**0.0001**	0.454000
original_ngtdm_Coarseness	405.28	0.621593	**0.0001**	**0.507000**
*Ex vivo* CT *versus* *in vivo* CT (original)				
original_gldm_DependenceNonUniformity	450.22	0.362503	0.7688	0.012000
original_gldm_GrayLevelNonUniformity	316.28	0.368821	0.6634	0.111000
original_glrlm_GrayLevelNonUniformity	559.75	0.340391	1.0000	0.154000
original_ngtdm_Coarseness	142.47	0.315376	1.0000	0.400000
*Ex vivo* CT *versus* micro-CT				
original_gldm_DependenceNonUniformity	223.42	0.782132	**0.0001**	0.399000
original_gldm_GrayLevelNonUniformity	241.99	0.578778	**0.0005**	0.355000
original_glrlm_GrayLevelNonUniformity	471.07	0.667554	**0.0001**	**0.570000**
original_ngtdm_Coarseness	848.18	0.625947	**0.0001**	0.410000
Micro-CT *versus* *in vivo* CT (subsampled)				
original_gldm_DependenceNonUniformity	642.00	0.701987	**0.0001**	0.328000
original_gldm_GrayLevelNonUniformity	328.50	0.771479	**0.0001**	**0.523000**
original_glrlm_GrayLevelNonUniformity	292.79	0.783694	**0.0001**	**0.685000**
original_ngtdm_Coarseness	405.15	0.791642	**0.0001**	**0.641000**
Micro-CT *versus* *in vivo* CT (original)				
original_gldm_GrayLevelNonUniformity	451.67	0.467901	**0.0198**	0.255000
original_gldm_DependenceNonUniformity	186.75	0.425697	0.0803	0.221000
original_glrlm_GrayLevelNonUniformity	317.15	0.435233	0.0594	0.360000
original_ngtdm_Coarseness	176.29	0.425513	0.0808	0.290000

The table reports the percentage change (%Change), Spearman’s correlation coefficient (ρ) with its *p*-value, and the intraclass correlation coefficient (ICC) for the texture features showing the highest reproducibility and correlation between modalities. Statistically significant Spearman's *p*-values (*p* < 0.05) and ICC values ≥ 0.50, indicating at least moderate agreement, are highlighted in bold

*CT* Computed tomography, *Micro-CT* Micro-computed tomography, *Original* Whole-tumor segmentation, *Subsampled* Volume-matched segmentation

**Fig. 4:**
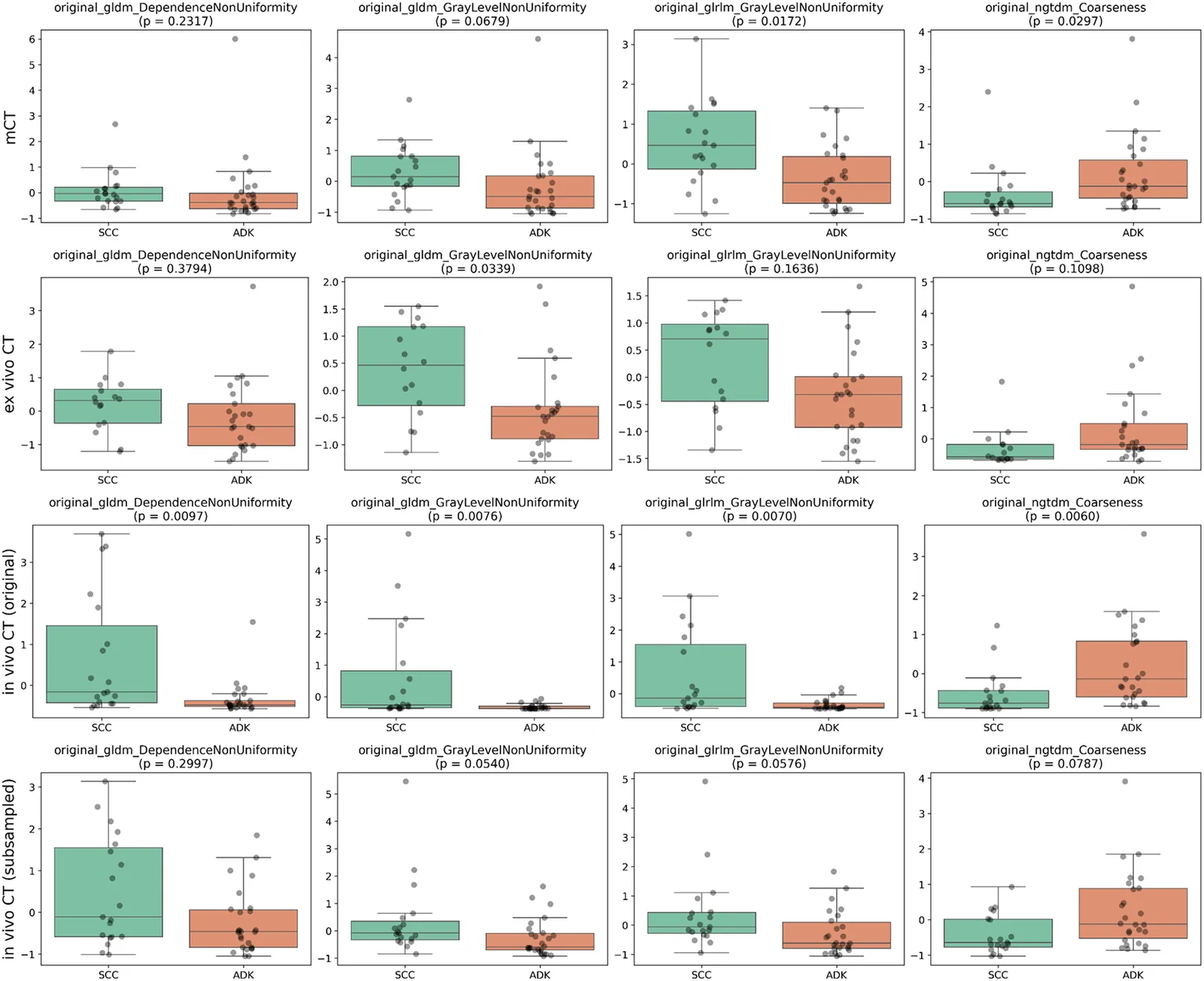
Univariate box plots comparing the distributions of selected radiomic features between clinical groups (ADK and SCC) across imaging modalities. Feature values are shown after z-score normalization. Each subplot reports the *p*-value (Bonferroni-corrected) from the Mann–Whitney U test. Rows correspond to different acquisition modalities (micro-CT, *ex vivo* CT, *in vivo* CT original, and *in vivo* CT subsampled), while columns represent distinct radiomic features. Box plots display the median, interquartile range, and individual data points. Gray-Level Non-Uniformity and Coarseness features show significant differences between groups in specific imaging conditions. ADK, Adenocarcinoma; CT, Computed tomography; GLDM, Gray-level dependence matrix; GLRLM, Gray-level run length matrix; Micro-CT, Micro-computed tomography; NGTDM, Neighboring gray tone difference matrix; Original, Whole-tumor segmentation; SCC, Squamous cell carcinoma; Subsampled, Volume-matched segmentation

Distributions of the most stable texture features between ADK and SCC with Mann–Whitney *U* test *p*-values are shown in Fig. 4.

**Fig. 5:**
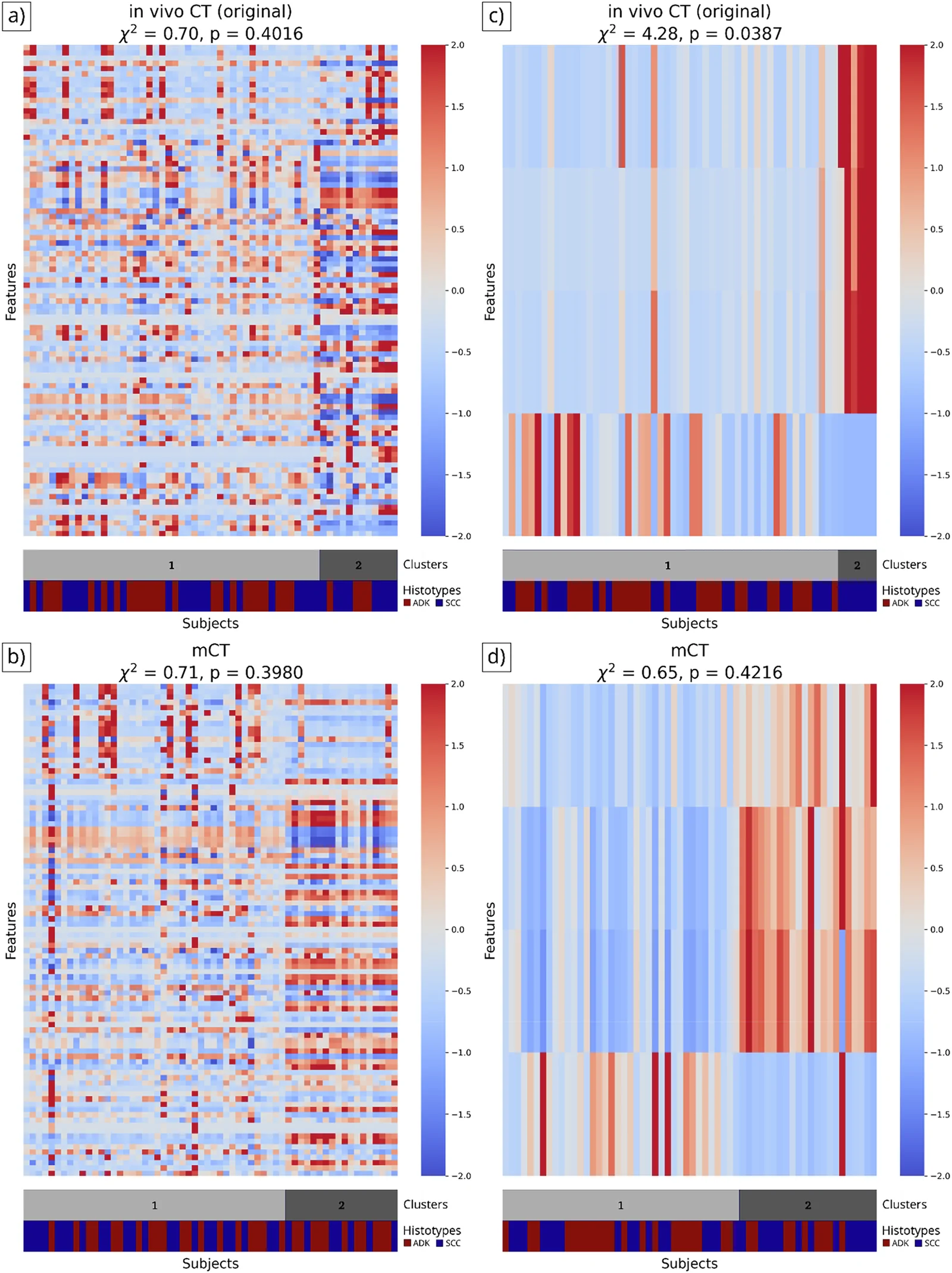
Radiomic clustering heatmaps showing the unsupervised grouping of patients based on radiomic texture features. **a**, **b** The clustering results for CT and micro-CT datasets, respectively, including all extracted features. **c**, **d** The clustering restricted to the subset of significant texture features, respectively, for CT and micro-CT datasets. Rows correspond to radiomic features, while columns represent individual patients. Color intensity reflects *z*-scored feature values, and the derived cluster assignments are shown below each heatmap. The association between cluster membership and histotype is reported using the χ² test, with *p*-values indicated in each panel. The clustering patterns suggest that subsets of texture features may partially capture histotype-related heterogeneity across imaging modalities. ADK, Adenocarcinoma; CT, Computed tomography; Micro-CT, Micro-computed tomography; Original, Whole-tumor segmentation; SCC, Squamous cell carcinoma

Associations between unsupervised clusters (derived from all features or only selected features) and histotypes were assessed (Fig. 5): (1) for the *in vivo* CT all features approach, χ² = 0.70 (*p* = 0.402); (2) for the micro-CT all features approach χ² = 0.71 (*p* = 0.398); (3) for the *in vivo* CT selected features approach χ² = 4.28 (*p* = 0.039) and (4) for the micro-CT selected features approach χ² = 0.65 (*p* = 0.422).

## Discussion

The literature has already highlighted the potential of micro-CT as a complementary tool for lung cancer characterization, providing a direct link between radiological features and histopathological findings [21]. In a preclinical setting, Brown et al demonstrated that harmonized micro-CT and cone-beam CT acquisitions can provide robust, tissue-specific radiomic features [15]. However, few studies have investigated the applicability of radiomic methods to micro-CT images and, most importantly, evaluated their applicability to clinical CT. In this context, the reproducibility of radiomic features represents not only a technical prerequisite but is also related to their biological and prognostic relevance. This was demonstrated by Parmar et al, who showed that the most stable features tend to possess the greatest discriminative and predictive power [22].

Regarding the effect of normalization and volume matching, we note that the analysis of concordance between the different acquisition modalities showed that intensity normalization and volume matching are important steps to improve radiomic feature repeatability. All reproducibility metrics were higher in the normalized condition. Consistent with previous findings by Shafiq-ul-Hassan et al (2018), normalization significantly reduced the percentage variability of first-order features, with the average variation decreasing from over 1,200% (non-normalized dataset) to approximately 320% (normalized dataset) [23].

Median ICC increased from ≈0.01–0.12 (non-normalized) to ≈0.20–0.23 (normalized), and the percentage of features with ICC > 0.5 rose from less than 5% to 12–18%. Spearman’s correlation coefficients were higher after normalization (ρ ≈ 0.20 *versus* 0.10 in non-normalized data), and the number of significantly correlated features increased. This effect is expected, since first-order features describe histogram statistics and are strongly influenced by the gray scale and intensity range, which are not comparable between images of different resolution and contrast.

Similar to the normalized dataset, the use of subsampled ROIs resulted in slightly higher ICC and correlation values compared with whole-tumor ROIs, increasing the significance percentages of the features (Spearman) by approximately 8–10% both in the comparison between *ex vivo* CT and *in vivo* CT and in the comparison between micro-CT and *in vivo* CT. Although the improvement was modest, this confirms that many radiomic features depend on ROI size and shape, and that reduction to a comparable volume allows for a more accurate comparison between modalities. Indeed, Shafiq-ul-Hassan et al (2017) report that many commonly used features (*e.g*., Coarseness, GrayLevelNonUniformity, Energy, RunLenghtNonUniformity) show a strong intrinsic dependence on voxel size and ROI volume [24]. The most repeatable setting was observed in the normalized dataset with subsampled ROI, which showed better metrics both for the comparison between *ex vivo* CT *versus* subsampled *in vivo* CT and for micro-CT *versus* subsampled *in vivo* CT. In both comparisons, approximately 21.51% of features showed a significantly correlated Spearman’s ρ, and over 17% of features had an ICC > 0. 5, while average ICC across all extracted features remained low (≈0.20) and was still above 0.60 for first-order features. In contrast, in non-normalized datasets, the average ICCs are reduced to 0.08–0.12, with less than 5% of features showing “moderate agreement” (ICC > 0.5). Notably, texture features still exhibited large magnitude differences across modalities (high %Change), indicating substantial residual variability despite normalization. Overall, these results indicate a relative improvement relatable to intensity harmonization and volume matching but also highlight that only a small subset of radiomic features can be considered acceptably stable across scales and under harmonized settings. Our conclusions are consistent with those reported in a large systematic review by Reiazi et al, who identified specific parameters (*e.g*., voxel size, slice thickness, and reconstruction kernel) as the most disruptive factors affecting radiomic robustness [25].

Literature has widely investigated the link between texture features and lung cancer histology. Aerts et al demonstrated that features related to intratumoral heterogeneity (*e.g*., GrayLevelNonUniformity and DependenceNonUniformity) are strongly associated with tumor phenotype [26]. Among the features tested, four proved to be consistently repeatable based on the significance of Spearman’s test in multiple comparisons and analysis conditions (Table 2 and Fig. 3). Their geometric and biological significance is summarized in Table 5 in the Supplementary Materials. In general, these features derive from dependency or run-length matrices and quantify the non-uniformity of gray levels and the local structural variability of the image. In biological terms, these texture features have been associated with tumor heterogeneity and variations in tissue architecture (including differences in cellularity) in prior radiomics literature [27]. The preserved pattern and correlation of these features on micro-CT suggest that they may represent candidate radiomic features with consistent cross-modality behavior, maintaining similar trends across images acquired at different resolution scales and protocols.

Feature distributions (Fig. 4) showed that, in *in vivo* conventional CT without subsampling, the four selected features significantly differentiate NSCLC histotypes (ADK *versus* SCC), with higher values in SCC (*p* < 0.01), except for original_ngtdm_Coarseness that tended to be higher in ADK. In the *in vivo* subsampled setting, the same directional trends were generally maintained, although statistical significance was reduced (*p* ≈ 0.05–0.08 for some comparisons and non-significant for others). Across modalities, including micro-CT, the selected features displayed broadly comparable trend patterns between histotypes. This suggests that the structural characteristics of the tumor (*e.g*., cellularity, intrinsic heterogeneity, *etc*.) are perceived by images at different resolutions, supporting the hypothesis that a subset of texture features may capture underlying structural heterogeneity.

Unsupervised clustering (Fig. 5) showed that only models based on the selected features are able to produce distributions significantly associated with histotype, while models using all features were not significant. This result supports the hypothesis that feature stability is a useful parameter not only for technical repeatability but also for clinical-biological oriented radiomic models. The most informative features maintain the same variation trend and the same discriminating capacity at different spatial scales, suggesting a possible correlation with tissue microarchitecture. However, it should be noted that the biological associations reported from histotype and clustering analyses are preliminary and exploratory. Building on these findings, future work will aim to validate the identified stable features in independent external datasets to assess their generalizability across scanners, centers, and acquisition protocols.

This study has limitations: (1) small sample size and single-center study, independent multi-center external validation will be required in future work; (2) micro-CT specimens represented only partial tumor volumes compared with whole-tumor *in vivo* CT; (3) subsampling may have introduced spatial sampling bias; (4) *in vivo* to *ex vivo* alignment relied on axial reorientation only, which may not fully account for local deformations affecting texture features; (5) segmentations were performed by a single operator and formal inter-observer variability could not be assessed within the present study design; (6) the lack of an external dataset for independent validation; (7) heterogeneous acquisition protocols for *in vivo* CT scans; (8) absence of direct quantitative histopathological validation (*e.g*., cellularity, stromal fraction, necrosis), future studies may integrate quantitative pathology to provide direct validation.

In conclusion, this study systematically explored radiomic feature behavior across *in vivo* CT and micro-CT imaging domains, contributing to efforts to bridge radiological and histopathological scales. Although overall reproducibility across acquisition settings was limited, intensity normalization and volume matching reduced variability for specific feature classes. Notably, a subset of texture-based features showed relatively consistent patterns across resolutions, suggesting that certain aspects of tumor structural heterogeneity may be captured in a scale-aware manner.

These findings support the use of micro-CT as an exploratory platform for investigating cross-scale radiomic stability and may inform future studies aiming to develop more robust and biologically interpretable radiomic models.

## Supplementary information

**Table 1**. List of IBSI compliant extracted radiomic features: 18 first-order features, 14 shape-based feautures, and x textural features (16 features based on Gray Level Size Zone Matrix (GLSZM), 24 Gray Level Co-occurrence Matrix (GLCM) features, 14 Gray Level Dependence Matrix (GLDM) and 5 Neighboring Gray Tone Difference Matrix (NGTDM)). **Table 2**. Demographic and pathological characteristics of the study cohort. The table summarizes the main clinical and histopathological features of the 60 patients included in the study. Data are reported as mean ± standard deviation (SD) and range for continuous variables, and as percentage (number) for categorical variables. Others pathological dominant pattern includes pagetoid, sarcomatoid, heterogeneous and undefined patterns. Tumors were evenly distributed between adenocarcinoma and squamous cell carcinoma histotypes. The majority of patients presented T1–T2 lesions, absence of nodal involvement, and no distant metastases. **Table 3**. Summary of results for non-normalized datasets. The table reports comparisons between *ex vivo* CT vs *in vivo* CT, mCT vs *ex vivo* CT, and mCT vs *in vivo* CT, considering CT *in vivo* both with whole-tumour ROIs (Original) and with volume-matched cylindrical ROIs (Subsampled). For each comparison, results are provided for all features combined, as well as for the main families of features analysed (First Order and Texture). Quantitative parameters are expressed as mean ± standard deviation (minimum; maximum) for continuous variables and as percentage (absolute number) for categorical outcomes. %Change represents the average absolute relative variation in feature values across modalities, Spearman (ρ) indicates the correlation coefficient; Spearman (*p* < 0.05), Spearman (*p* < 0.01) and Spearman (*p* < 0.001) refer to the percentage (and number) of features showing statistically significant correlations at the respective thresholds; ICC corresponds to the intraclass correlation coefficient. **Table 4**. Metrics of the most stable texture features across modality comparisons in NonNormalized datasets. The table reports the percentage change (%Change), Spearman’s correlation coefficient (ρ) with its *p*-value, and the intraclass correlation coefficient (ICC) for the texture features showing the highest reproducibility and correlation between modalities. **Table 5**. Meaning and biological interpretation of the most repeatable radiomic features. **Figure 1**. Image preprocessing and segmentation corrections. (a) Correction of segmentation errors in mCT images. The semi-automatic region-growing segmentation occasionally left small internal voids that interfered with feature extraction; these artefacts were corrected to obtain continuous tumour masks. Some examples are reported in the image. (b) In conventional CT images, the entire tumour volume was segmented, whereas mCT scans were obtained from surgical specimens that did not include the whole lesion. Therefore, to achieve volumetric comparability, the CT segmentations were undersampled to obtain subregions with volumes similar to the corresponding mCT specimens. The subregions were localized within the *in vivo* CT images at the anatomical site where the surgical specimen was obtained, with minimal spatial approximation. (c) Spearman correlation analysis demonstrated a strong volumetric agreement (ρ ≈ 1, *p* < 0.001) between the resampled CT and mCT segmentations after subregion matching, confirming geometric consistency between modalities. These preprocessing steps ensured consistent voxel geometry across all imaging modalities, enabling direct quantitative comparison of radiomic features between microCT, CT *ex vivo*, and CT *in vivo* datasets. **Figure 2**. Scatter plot matrices showing pairwise correlations between radiomic features extracted from different imaging modalities without gray-level normalization (Non-normalized dataset). The comparisons include *ex vivo* CT vs. *in vivo* CT (subsampled), *ex vivo* CT vs. *in vivo* CT (original), mCT vs. *ex vivo* CT, mCT vs. *in vivo* CT (subsampled), and mCT vs. *in vivo* CT (original). Red points indicate pairs of features that are significantly correlated (Spearman’s rank correlation coefficient, *p* < 0.05). The plots demonstrate that many first-order features remain consistent across imaging conditions, whereas certain texture features exhibit higher concordance particularly in the comparisons performed after volume matching. **Figure 3**. Bland–Altman plots illustrating the agreement between radiomic features extracted from different imaging modalities after gray-level normalization (normalized dataset). The comparisons include *ex vivo* CT vs. *in vivo* CT (subsampled), *ex vivo* CT vs. *in vivo* CT (original), mCT vs. *ex vivo* CT, mCT vs. *in vivo* CT (subsampled), and mCT vs. *in vivo* CT (original). **Figure 4**. Bland–Altman plots illustrating the agreement between radiomic features extracted from different imaging modalities without gray-level normalization (Non-normalized dataset). The comparisons include *ex vivo* CT vs. *in vivo* CT (subsampled), *ex vivo* CT vs. *in vivo* CT (original), mCT vs. *ex vivo* CT, mCT vs. *in vivo* CT (subsampled), and mCT vs. *in vivo* CT (original). **Figure 5**. Bar plots of Spearman correlation coefficients (ρ) between radiomic features extracted from different imaging modalities after gray-level normalization (normalized dataset). Panels show: (a) *ex vivo* CT vs. *in vivo* CT (subsampled), (b) *ex vivo* CT vs. *in vivo* CT (original), (c) mCT vs. *in vivo* CT (subsampled), (d) mCT vs. *in vivo* CT (original), and (e) mCT vs. *ex vivo* CT. Bars represent individual radiomic features, colored according to the level of statistical significance (*p* < 0.05, *p* < 0.01, *p* < 0.001). Positive correlations are shown in blue, negative in red. The plots indicate that several first-order features exhibit strong and significant correlations across imaging conditions, whereas texture-related features display greater variability, particularly in comparisons involving unaligned or non–volume-matched datasets. **Figure 6**. Bar plots of Spearman correlation coefficients (ρ) between radiomic features extracted from different imaging modalities without gray-level normalization (Non-normalized dataset). Panels show: (a) *ex vivo* CT vs. *in vivo* CT (subsampled), (b) *ex vivo* CT vs. *in vivo* CT (original), (c) mCT vs. *in vivo* CT (subsampled), (d) mCT vs. *in vivo* CT (original), and (e) mCT vs. *ex vivo* CT. Bars represent individual radiomic features, colored according to the level of statistical significance (*p* < 0.05, *p* < 0.01, *p* < 0.001). Positive correlations are shown in blue, negative in red. The plots indicate that several first-order features exhibit strong and significant correlations across imaging conditions, whereas texture-related features display greater variability, particularly in comparisons involving unaligned or non–volume-matched datasets.

## Data Availability

The datasets used and analyzed during the current study are available from the corresponding author on reasonable request.

## Abbreviations

ADK: Adenocarcinoma
CT: Computed tomography
GLDM: Gray-level dependence matrix
GLRLM: Gray-level run length matrix
IBSI: Image Biomarker Standardisation Initiative
ICC: Intraclass correlation coefficient
Micro-CT: Micro-computed tomography
NGTDM: Neighboring gray tone difference matrix
NSCLC: Non-small-cell lung cancer
ROI: Region of interest
SCC: Squamous cell carcinoma
