# Supplementary material for: Bridging radiomics and histopathology: cross-modality consistency of radiomic features from CT to micro-CT in NSCLC
Source: Eur Radiol Exp. 2026 Oct 7;10:150. doi: 10.1186/s41747-026-00780-5 (PMC13646218; doi:10.1186/s41747-026-00780-5)
Supplement: Supplementary file 1 [file 41747_2026_780_MOESM1_ESM.pdf]

SUPPLEMENTARY MATERIALS

Bridging Radiomics and Histopathology: Cross-Modality Consistency of Radiomic Features from CT to Micro-CT in NSCLC

Intensity (first-order)
Entropy
Uniformity
Kurtosis
Minimum
Skewness
Range
90 Percentile
Maximum
Mean
Variance
Total Energy
Energy
Median
**Shape**
Elongation
Maximum 2D Diameter Column
Major Axis Lenght
Minor Axis Lenght
Maximum 3D Diameter
Maximum 2D Diameter Slice
Maximum 2D Diameter Row
Sphericity
Surface Area
Flatness
Least Axis Lenght
Mesh Volume
Voxel Volume
Surface Volume Ratio
**Texture (GLSZM)**
Zone Entropy
Small Area Emphasis
Size Zone Non Uniformity Normalized
Zone Percentage
Gray Level Variance
Small Area Low Gray Level Emphasis
Low Gray Level Zone Emphasis
Gray Level Non Uniformity Normalized
High Gray Level Zone Emphasis
Small Area High Gray Level Emphasis
Size Zone Non Uniformity
Gray Level Non Uniformity
Large Area High Gray Level Emphasis
Large Area Emphasis
Large Area Low Gray Level Emphasis
Zone Variance
**Texture (GLCM)**
Idmn
Idn
Imc2
Sum Entropy
Joint Entropy
MCC
Difference Entropy
Joint Average
Sum Average
Difference Average
Correlation
Id

Imc1
Difference Variance
Contrast
Inverse Variance
Idm
Autocorrelation
Sum Squares
Cluster Tendency
Maximum Probability
Texture (GLDM)
Dependence Entropy
Dependence Non Uniformity Normalized
Small Dependence Emphasis
Small Dependence Low Gray Level Emphasis
High Gray Level Emphasis
Large Dependence High Gray Level Emphasis
Small Dependence High Gray Level Emphasis
Gray Level Variance
Dependence Variance
Low Gray Level Emphasis
Large Dependence Emphasis
Large Dependence Low Gray Level Emphasis
Dependence Non Uniformity
Gray Level Non Uniformity
Texture (GLRLM)
Run Entropy
Short Run Emphasis
Run Percentage
Run Length Non Uniformity Normalized
Gray Level Variance
Short Run Low Gray Level Emphasis
Long Run High Gray Level Emphasis
Low Gray Level Run Emphasis
High Gray Level Run Emphasis
Short Run High Gray Level Emphasis
Gray Level Non Uniformity Normalized
Long Run Emphasis
Run Variance
Long Run Low Gray Level Emphasis
Run Length Non Uniformity
Gray Level Non Uniformity
Texture (NGTDM)
Strenght
Coarsness
Complexity
Contrast
Busyness

Table 1. List of IBSI compliant extracted radiomic features: 18 first-order features, 14 shape-based feautures, and x textural features (16 features based on Gray Level Size Zone Matrix (GLSZM), 24 Gray Level Co-occurrence Matrix (GLCM) features, 14 Gray Level Dependence Matrix (GLDM) and 5 Neighboring Gray Tone Difference Matrix (NGTDM)).

Variable	Category	Value
Age (years)	Mean ± SD (range)	74.4 ± 7.7years (54–91years)
Tumour diameter (cm)	Mean ± SD (range)	2.97 cm ± 1.58cm (1.0–7.5cm)
Time between CT and surgery (days)	Mean ± SD (range)	73.5 ± 67.3days (2–448days).
Sex	M	65% (n=39)
	F	35% (n=21)
Tumour Location	SLL	28% (n=17)
	SRL	27% (n=16)
	IRL	17% (n=10)
	ILL	17% (n=10)
	ML	12% (n=7)
Pathological dominant pattern	Necrosis	28.3% (n=17)
	Lepidic	21.7% (n=13)
	Acinar	15.0% (n=9)
	Solid	11.7% (n=7)
	Others	23.3% (n=14)
Pleural invasion	No	73.3% (n=44)
	Yes	26.7% (n=16)
Vascular invasion	No	41.7% (n=25)
	Yes	58.3% (n=35)
Distant alveolar growth	No	80.0% (n=48)
	Yes	20.0% (n=12)
Positive bronchial margins	No	98.3% (n=59)
	Yes	1.7% (n=1)
Histological Grading	G2	73.3% (n=44)
	G3	25.0% (n=15)
	G1	1.7% (n=1)
Histotype	SCC	50.0% (n=30)
	ADK	50.0% (n=30)
T	1b	31.7% (n=19)
	3	20% (n=12)
	2a	18.3% (n=11)
	1c	15.0% (n=9)
	2b	10.0% (n=6)
	1a	5.0% (n=3)
N	0	85.0% (n=51)
	1	11.7% (n=7)
	2	3.3% (n=2)
M	0	96.7% (n=58)
	1	3.3% (n=2)

Table 2. Demographic and pathological characteristics of the study cohort.

The table summarizes the main clinical and histopathological features of the 60 patients included in the study. Data are reported as mean ± standard deviation (SD) and range for continuous variables, and as percentage (number) for categorical variables. Others pathological dominant pattern includes pagetoid, sarcomatoid, heterogeneous and undefined patterns. Tumors were evenly distributed between adenocarcinoma and squamous cell carcinoma histotypes. The majority of patients presented T1–T2 lesions, absence of nodal involvement, and no distant metastases.

Parameter	Group	Comparisons				
		CT ex vivo vs CT in vivo Subsampled	CT ex vivo vs CT in vivo Original	mCT vs CT ex vivo	mCT vs CT in vivo Subsampled	mCT vs CT in vivo Original
**%Change**	All Features	**596,50±1699,56** (132,89;15817,31)	**399,80±350,67** (118,87;2862,59)	**530,19±586,38** (124,12;3599,39)	**543,27±879,39** (104,13;6602,81)	**507,30±1063,37** (100,71;8351,70)
	First Order	**1223,90±3647,57** (165,80;15817,31)	**367,13±186,15** (152,39;792,27)	**615,36±735,38** (191,90;3410,91)	**564,46±715,59** (165,02;3243,11)	**846,90±1881,05** (232,22;8351,70)
	Texture	**445,92±664,90** (132,89;5053,93)	**407,64±380,26** (118,87;2862,59)	**509,75±548,68** (124,12;3599,39)	**538,19±918,51** (104,13;6602,81)	**425,80±747,09** (100,71;6372,01)
**Spearman (ρ)**	All Features	**0,12±0,19** (-0,26;0,66)	**0,13±0,17** (-0,37;0,40)	**0,10±0,19** (-0,22;0,78)	**0,09±0,23** (-0,27;0,82)	**0,05±0,18** (-0,29;0,48)
	First Order	**0,04±0,07** (-0,08;0,19)	**0,22±0,09** (0,09;0,36)	**0,14±0,12** (-0,13;0,31)	**0,00±0,10** (-0,17;0,22)	**-0,06±0,11** (-0,25;0,17)
	Texture	**0,14±0,21** (-0,26;0,66)	**0,10±0,17** (-0,37;0,40)	**0,10±0,20** (-0,22;0,78)	**0,11±0,24** (-0,27;0,82)	**0,08±0,18** (-0,29;0,48)
**Spearman (p<0.05)**	All Features	8,60%(8)	0,00%(0)	7,53%(7)	8,60%(8)	2,15%(2)
	First Order	0,00%(0)	0,00%(0)	0,00%(0)	0,00%(0)	0,00%(0)
	Texture	10,66%(8)	0,00%(0)	9,33%(7)	10,67%(8)	2,66%(2)
**Spearman (p<0.01)**	All Features	6,45%(6)	0,00%(0)	6,45%(6)	7,52%(7)	0,00%(0)
**Spearman (p<0.001)**	All Features	5,38%(5)	0,00%(0)	5,38%(5)	7,52%(7)	0,00%(0)
**ICC**	All Features	**0,12±0,15** (-0,18;0,52)	**0,11±0,17** (-0,28;0,50)	**0,08±0,15** (-0,21;0,57)	**0,08±0,19** (-0,29;0,70)	**0,01±0,16** (-0,33;0,42)
	First Order	**0,10±0,06** (0,01;0,22)	**0,23±0,18** (-0,06;0,50)	**0,15±0,13** (-0,07;0,44)	**0,01±0,11** (-0,11;0,23)	**-0,04±0,14** (-0,23;0,18)
	Texture	**0,13±0,16** (-0,18;0,52)	**0,08±0,15** (-0,28;0,40)	**0,07±0,15** (-0,21;0,57)	**0,09±0,20** (-0,29;0,70)	**0,02±0,17** (-0,33;0,42)
**ICC >0.5 (Moderate-Good-Excellent)**	All Features	3,23%(3)	0,00%(0)	1,08%(1)	4,30%(4)	0,00%(0)
	First Order	0,00%(0)	0,00%(0)	0,00%(0)	0,00%(0)	0,00%(0)
	Texture	4,00%(3)	0,00%(0)	1,33%(1)	5,33%(4)	0,00%(0)
**ICC >0.75 (Good-Excellent)**	All Features	0,00%(0)	0,00%(0)	0,00%(0)	0,00%(0)	0,00%(0)
	First Order	0,00%(0)	0,00%(0)	0,00%(0)	0,00%(0)	0,00%(0)
	Texture	0,00%(0)	0,00%(0)	0,00%(0)	0,00%(0)	0,00%(0)
**ICC >0.90 (Excellent)**	All Features	0,00%(0)	0,00%(0)	0,00%(0)	0,00%(0)	0,00%(0)
	First Order	0,00%(0)	0,00%(0)	0,00%(0)	0,00%(0)	0,00%(0)
	Texture	0,00%(0)	0,00%(0)	0,00%(0)	0,00%(0)	0,00%(0)

Table 3. Summary of results for non-normalized datasets. The table reports comparisons between ex vivo CT vs in vivo CT, mCT vs ex vivo CT, and mCT vs in vivo CT, considering CT in vivo both with whole-tumour ROIs (Original) and with volume-matched cylindrical ROIs (Subsampled). For each comparison, results are provided for all features combined, as well as for the main families of features analysed (First Order and Texture). Quantitative parameters are expressed as mean ± standard deviation (minimum; maximum) for continuous variables and as percentage (absolute number) for categorical outcomes. %Change represents the average absolute relative variation in feature values across modalities, Spearman (ρ) indicates the correlation coefficient; Spearman (p<0.05), Spearman (p<0.01) and Spearman (p<0.001) refer to the percentage (and number) of features showing statistically significant correlations at the respective thresholds; ICC corresponds to the intraclass correlation coefficient.

Dataset/Feature	Percentage Change	Spearman (ρ)	Spearman p-value	ICC
**ex vivo CT vs in vivo CT (subsampled)**				
original_gldm_DependenceNonUniformity	423,12	0,641832	**0,0001**	0,447000
original_gldm_GrayLevelNonUniformity	274,01	0,614820	**0,0001**	0,382000
original_glrlm_GrayLevelNonUniformity	185,4	0,655056	**0,0001**	0,455000
original_ngtdm_Coarseness	394,11	0,6211432	**0,0001**	**0,507000**
**ex vivo CT vs in vivo CT (original)**				
original_gldm_DependenceNonUniformity	447,71	0,362503	0,7687	0,012000
original_gldm_GrayLevelNonUniformity	310,48	0,368821	0,6634	0,111000
original_glrlm_GrayLevelNonUniformity	556,54	0,340391	1,0000	0,154000
original_ngtdm_Coarseness	142,58	0,315376	1,0000	0,400000
**ex vivo CT vs mCT**				
original_gldm_DependenceNonUniformity	223,79	0,782132	**0,0001**	0,399000
original_gldm_GrayLevelNonUniformity	242,07	0,578778	**0,0005**	0,355000
original_glrlm_GrayLevelNonUniformity	471,44	0,667554	**0,0001**	**0,570000**
original_ngtdm_Coarseness	905,36	0,625947	**0,0001**	0,410000
**m CT vs in vivo CT (subsampled)**				
original_gldm_DependenceNonUniformity	647,38	0,701987	**0,0001**	0,328000
original_gldm_GrayLevelNonUniformity	330,49	0,771596	**0,0001**	**0,523000**
original_glrlm_GrayLevelNonUniformity	293,47	0,783694	**0,0001**	**0,685000**
original_ngtdm_Coarseness	947,13	0,791642	**0,0001**	**0,641000**
**m CT vs in vivo CT (original)**				
original_gldm_GrayLevelNonUniformity	304,6	0,435233	**0,0198**	0,360000
original_gldm_DependenceNonUniformity	188,01	0,425697	0,0803	0,222000
original_glrlm_GrayLevelNonUniformity	441,86	0,467901	0,0594	0,255000
original_ngtdm_Coarseness	173,99	0,425513	0,0808	0,290000

Table 4. Metrics of the most stable texture features across modality comparisons in NonNormalized datasets. The table reports the percentage change (%Change), Spearman's correlation coefficient (ρ) with its p-value, and the intraclass correlation coefficient (ICC) for the texture features showing the highest reproducibility and correlation between modalities.

Feature name	Class/ Matrix	Geometrical meaning	Biological or structural interpretation	Bibliographical references
original_gldm_ DependenceNonUniformity (DNU)	GLDM – Gray Level Dependence Matrix	Measures the non-uniformity of dependent voxels. High values indicate marked variations in the sizes of dependency groups.	Structural heterogeneity: tissue with regions of varying compactness or intensity homogeneity shows high DNU. Intra-lesional heterogeneity or architectural variability.	Thibault et al., Pattern Recogn. Inf. Process. 2009; Zwanenburg et al., IBSI v2, 2020; Chen et al., Diagnostics 2021
original_gldm_ GrayLevelNonUniformity (GLN)	GLDM– Gray Level Dependence Matrix	Evaluates the non-uniformity of grey level distribution in dependent regions. Low values indicate uniform distribution.	Reflects the variety of density or attenuation of the tissue: low GLN is indicative of a more homogeneous texture.	Thibault et al., Pattern Recogn. Inf. Process. 2009; Chen et al., Diagnostics 2021; Shafiq-ul-Hassan et al., Sci Rep 2018
original_glrlm_ GrayLevelNonUniformity (GLNU)	GLRLM – Gray Level Run Length Matrix	Measures the variation in grey level frequency along runs (sequences of voxels with the same value). High values indicate that only a few grey levels dominate.	Reflects uniformity of tissue composition along contiguous structures: high GLNU indicates predominance of similar tissue components, whereas low GLNU suggests more mixed, heterogeneous tissue.	Galloway, Comput. Graph. Image Process. 1975; Aerts et al., Nat Commun 2014; Balagurunathan et al., Transl Oncol 2014
original_ngtdm_ Coarseness	NGTDM – Neighbouring Gray Tone Difference Matrix	It is the inverse of the sum of the differences between each voxel and the average of its neighbours. High values indicate minimal local variations (coarse texture), while low values indicate rapid variations (fine texture).	Represents the perceived graininess of the texture. A high Coarseness indicates more uniform patterns, while low values correspond to fine microstructures or inhomogeneous tissues.	Amadasun & King, IEEE Trans. Syst. Man Cybern. 1989; Tixier et al., J Nucl Med 2011; Cook et al., J Nucl Med 2013; Shafiq-ul-Hassan et al., Sci Rep 2018

Table 5. Meaning and biological interpretation of the most repeatable radiomic features

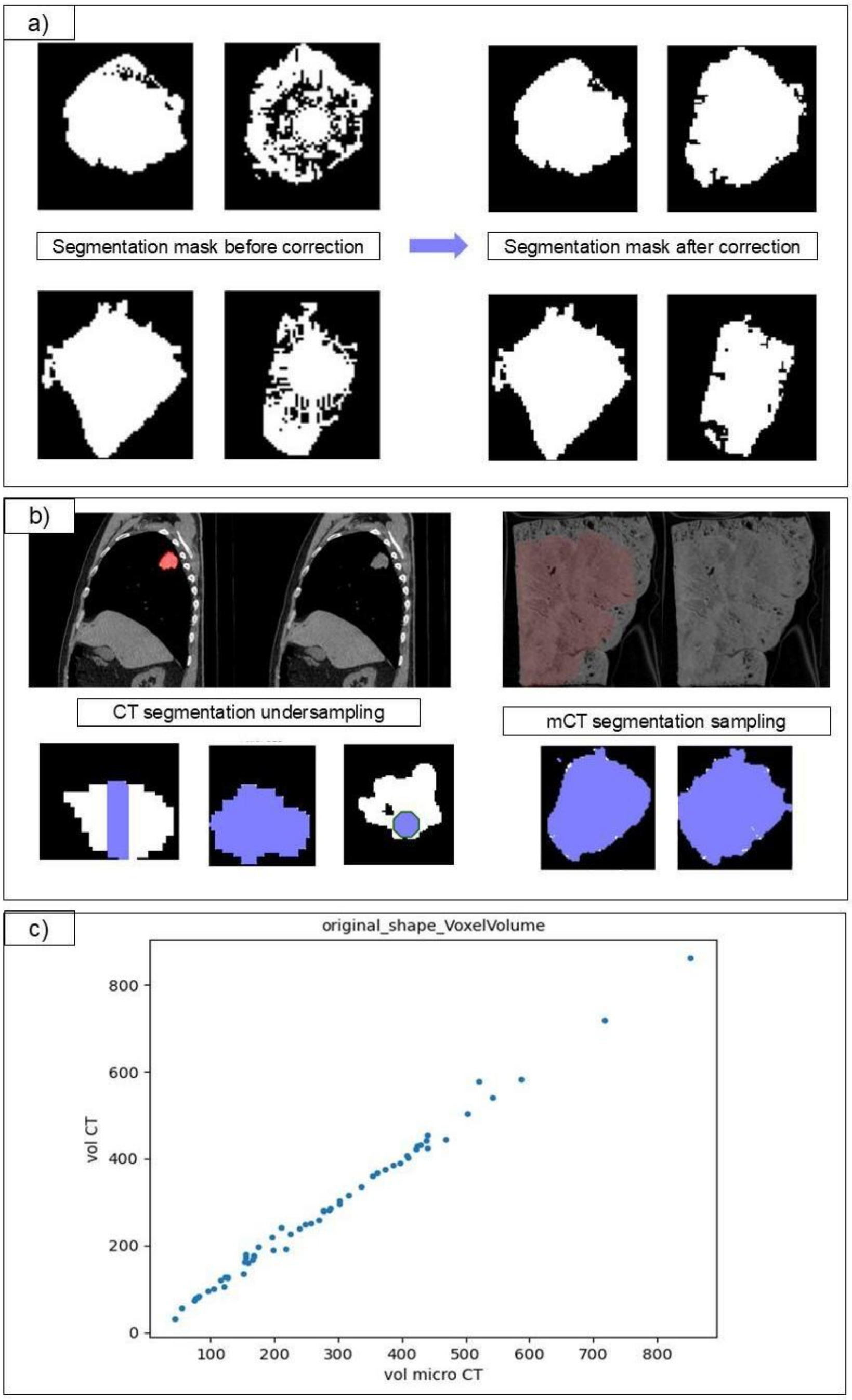

Figure 1. Image preprocessing and segmentation corrections.
(a) Correction of segmentation errors in mCT images. The semi-automatic region-growing segmentation occasionally left small internal voids that interfered with feature extraction; these artefacts were corrected to obtain continuous tumour masks. Some examples are reported in the image.
(b) In conventional CT images, the entire tumour volume was segmented, whereas mCT scans were obtained from surgical specimens that did not include the whole lesion. Therefore, to achieve volumetric comparability, the CT segmentations were undersampled to obtain subregions with volumes similar to the corresponding mCT specimens. The subregions were localized within the in vivo CT images at the anatomical site where the surgical specimen was obtained, with minimal spatial approximation.
(c) Spearman correlation analysis demonstrated a strong volumetric agreement ($\rho \approx 1$, $p < 0.001$) between the resampled CT and mCT segmentations after subregion matching, confirming geometric consistency between modalities.
These preprocessing steps ensured consistent voxel geometry across all imaging modalities, enabling direct quantitative comparison of radiomic features between microCT, CT ex vivo, and CT in vivo datasets.

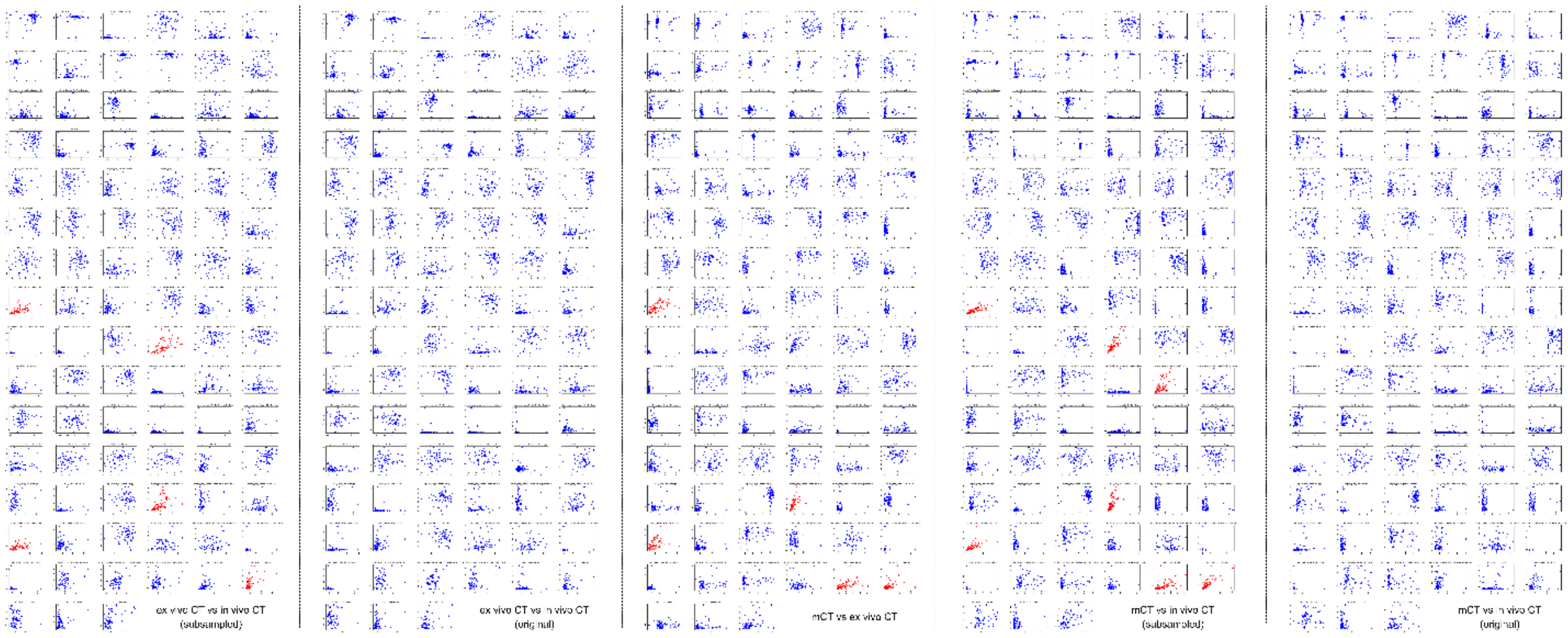

Figure 2. Scatter plot matrices showing pairwise correlations between radiomic features extracted from different imaging modalities without gray-level normalization (Non-normalized dataset). The comparisons include ex vivo CT vs. in vivo CT (subsampled), ex vivo CT vs. in vivo CT (original), mCT vs. ex vivo CT, mCT vs. in vivo CT (subsampled), and mCT vs. in vivo CT (original). Red points indicate pairs of features that are significantly correlated (Spearman's rank correlation coefficient, $p < 0.05$). The plots demonstrate that many first-order features remain consistent across imaging conditions, whereas certain texture features exhibit higher concordance particularly in the comparisons performed after volume matching.

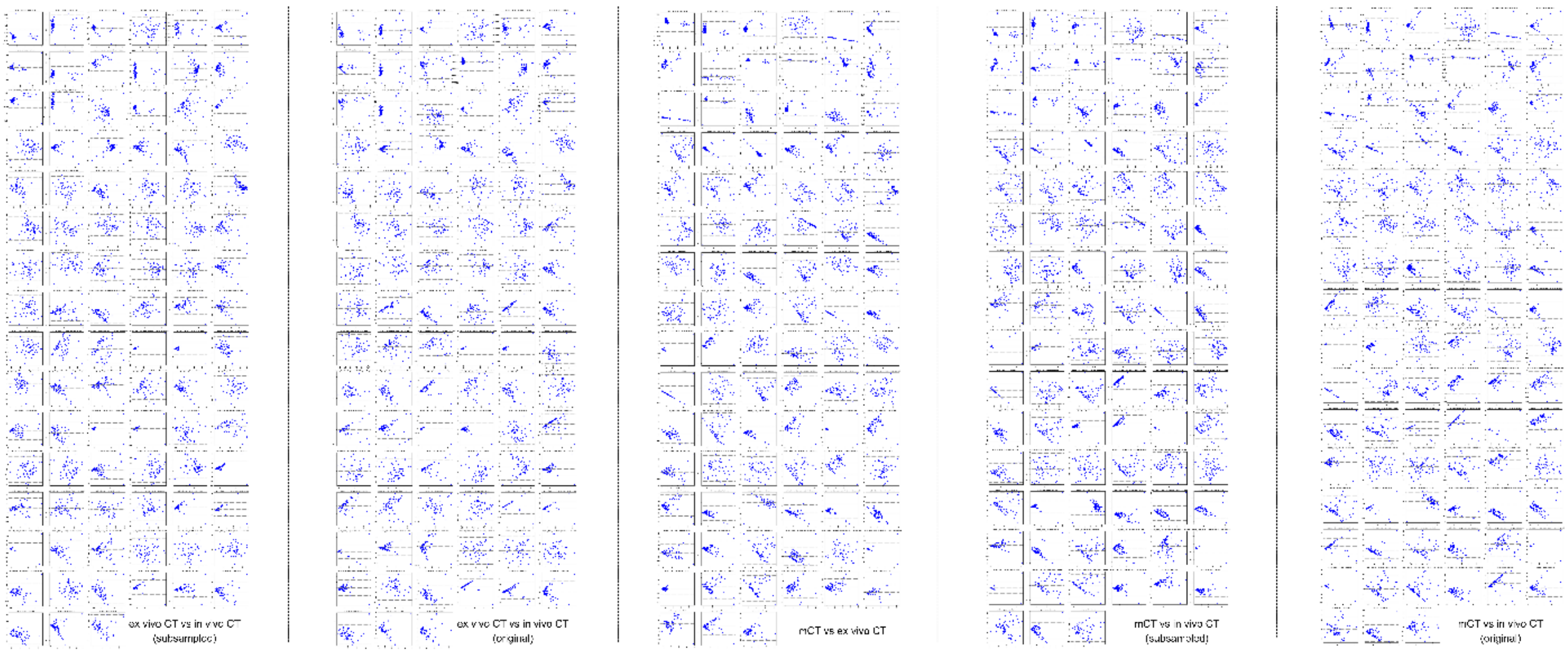

Figure 3. Bland–Altman plots illustrating the agreement between radiomic features extracted from different imaging modalities after gray-level normalization (normalized dataset). The comparisons include ex vivo CT vs. in vivo CT (subsampled), ex vivo CT vs. in vivo CT (original), mCT vs. ex vivo CT, mCT vs. in vivo CT (subsampled), and mCT vs. in vivo CT (original).

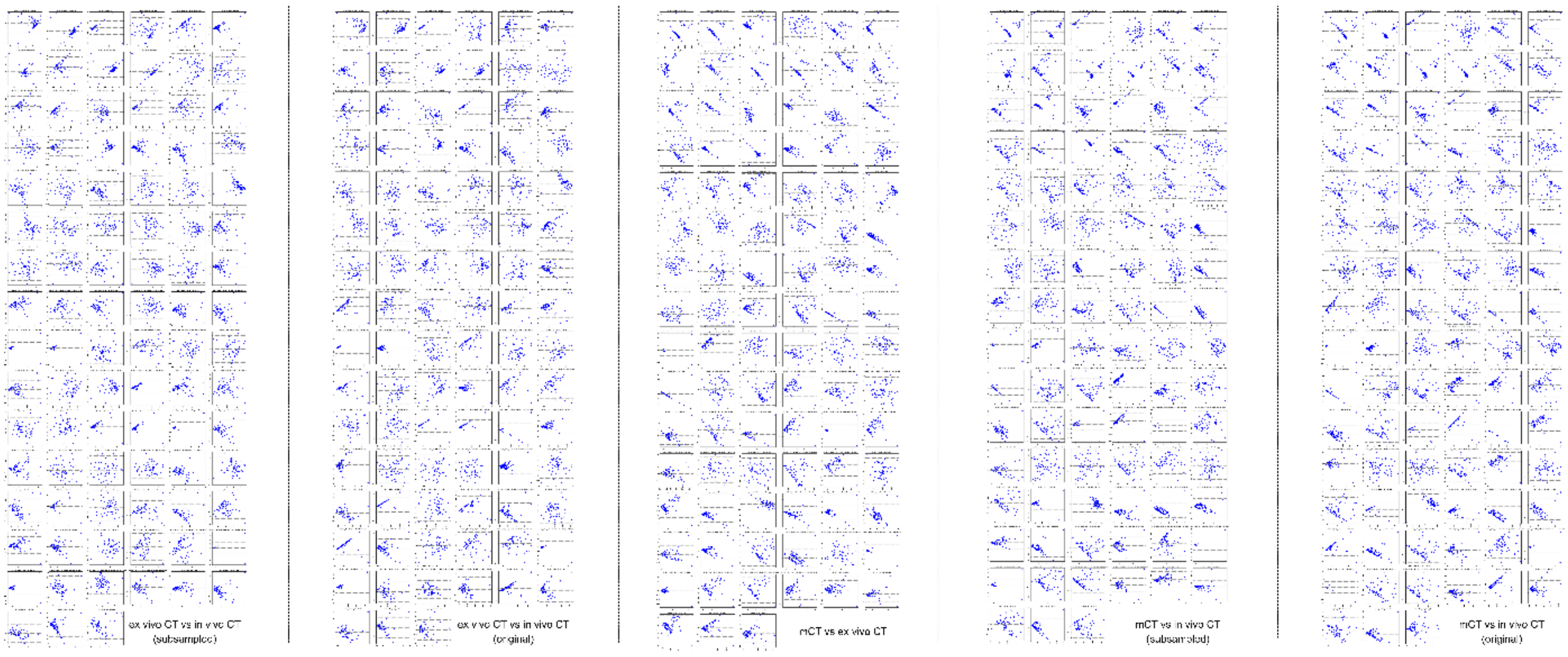

Figure 4. Bland–Altman plots illustrating the agreement between radiomic features extracted from different imaging modalities without gray-level normalization (Non-normalized dataset). The comparisons include ex vivo CT vs. in vivo CT (subsampled), ex vivo CT vs. in vivo CT (original), mCT vs. ex vivo CT, mCT vs. in vivo CT (subsampled), and mCT vs. in vivo CT (original).

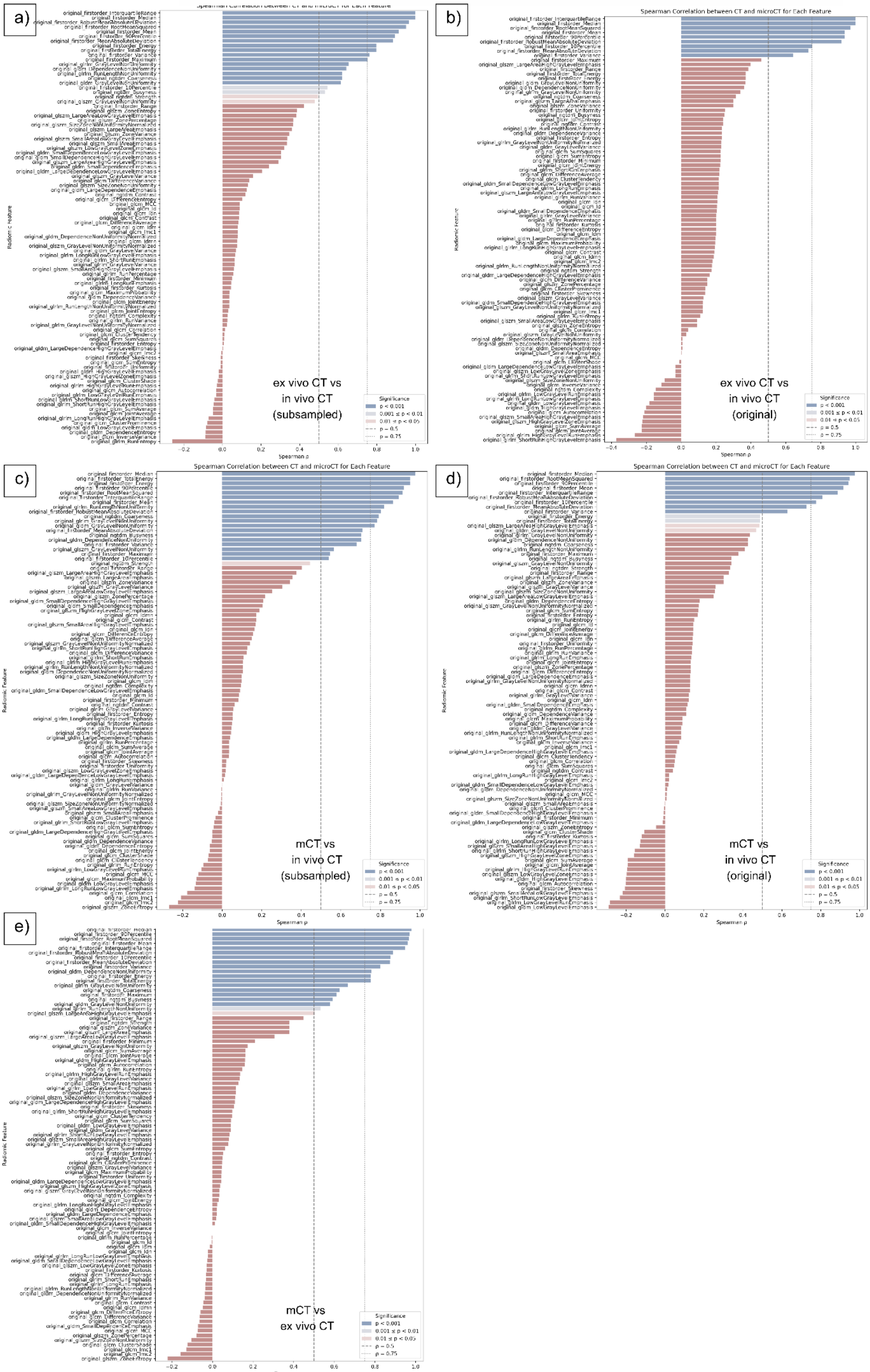
a)
ex vivo CT vs
in vivo CT
(subsampled)
b)
Spearman Correlation between CT and microCT for Each Feature
ex vivo CT vs
in vivo CT
(original)
c)
Spearman Correlation between CT and microCT for Each Feature
mCT vs
in vivo CT
(subsampled)
d)
Spearman Correlation between CT and microCT for Each Feature
mCT vs
in vivo CT
(original)
e)
mCT vs
ex vivo CT

Figure 5. Bar plots of Spearman correlation coefficients (ρ) between radiomic features extracted from different imaging modalities after gray-level normalization (normalized dataset). Panels show: (a) ex vivo CT vs. in vivo CT (subsampled), (b) ex vivo CT vs. in vivo CT (original), (c) mCT vs. in vivo CT (subsampled), (d) mCT vs. in vivo CT (original), and (e) mCT vs. ex vivo CT.
Bars represent individual radiomic features, colored according to the level of statistical significance ($p < 0.05$, $p < 0.01$, $p < 0.001$). Positive correlations are shown in blue, negative in red. The plots indicate that several first-order features exhibit strong and significant correlations across imaging conditions, whereas texture-related features display greater variability, particularly in comparisons involving unaligned or non–volume-matched datasets.

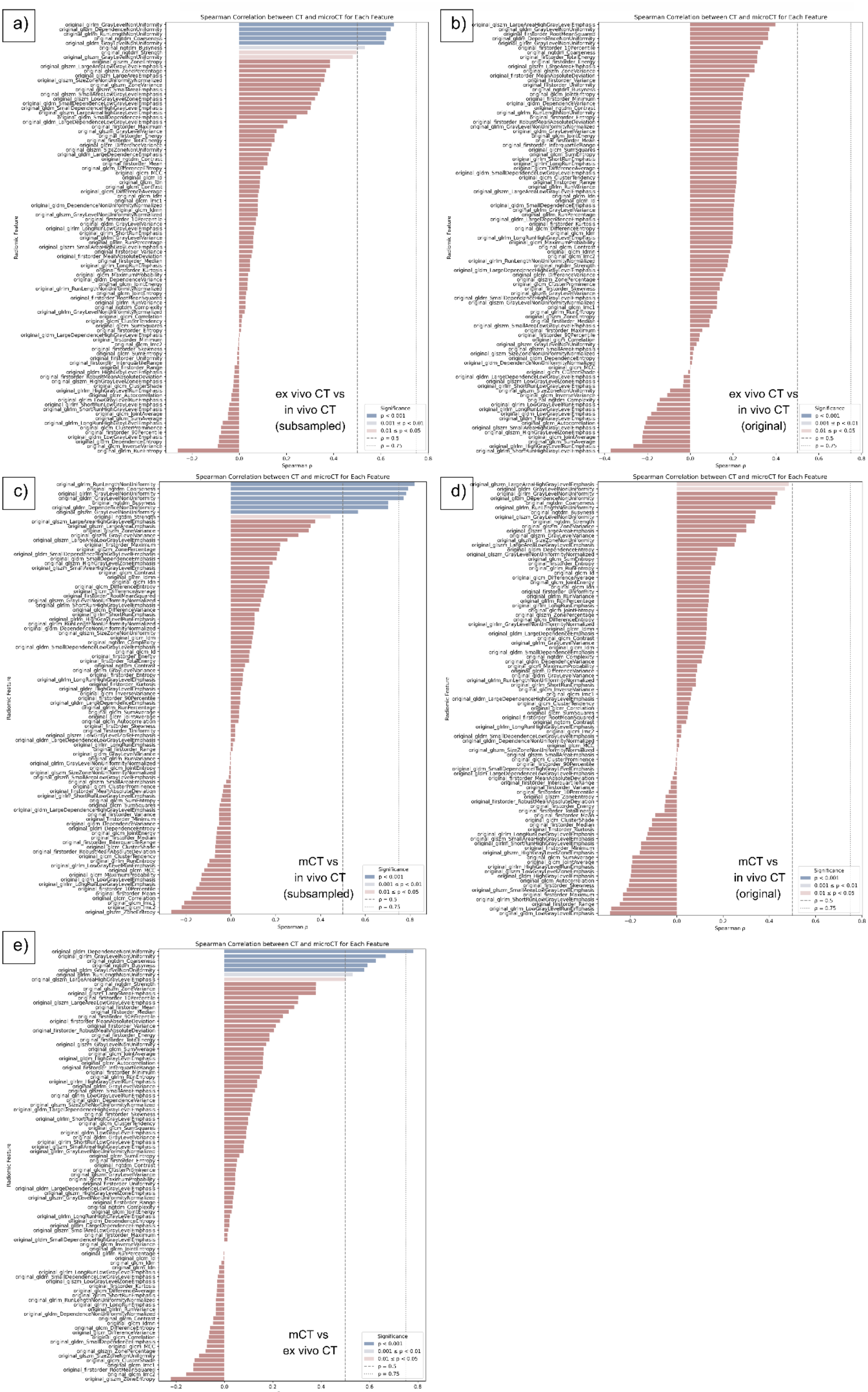
a)
Spearman Correlation between CT and microCT for Each Feature
ex vivo CT vs
in vivo CT
(subsampled)
b)
Spearman Correlation between CT and microCT for Each Feature
ex vivo CT vs
in vivo CT
(original)
c)
Spearman Correlation between CT and microCT for Each Feature
mCT vs
in vivo CT
(subsampled)
d)
Spearman Correlation between CT and microCT for Each Feature
mCT vs
in vivo CT
(original)
e)
Spearman Correlation between CT and microCT for Each Feature
mCT vs
ex vivo CT
Significance
p < 0.001
0.001 ≤ p < 0.01
0.01 ≤ p < 0.05
ρ = 0.5
ρ = 0.75
Radiomic Feature
Spearman ρ

Figure 6. Bar plots of Spearman correlation coefficients (ρ) between radiomic features extracted from different imaging modalities without gray-level normalization (Non-normalized dataset). Panels show: (a) ex vivo CT vs. in vivo CT (subsampled), (b) ex vivo CT vs. in vivo CT (original), (c) mCT vs. in vivo CT (subsampled), (d) mCT vs. in vivo CT (original), and (e) mCT vs. ex vivo CT.
Bars represent individual radiomic features, colored according to the level of statistical significance ($p < 0.05$, $p < 0.01$, $p < 0.001$). Positive correlations are shown in blue, negative in red. The plots indicate that several first-order features exhibit strong and significant correlations across imaging conditions, whereas texture-related features display greater variability, particularly in comparisons involving unaligned or non–volume-matched datasets.
